# Ultrafast terahertz emission from emerging symmetry-broken materials

**DOI:** 10.1038/s41377-023-01163-w

**Published:** 2023-06-01

**Authors:** Jacob Pettine, Prashant Padmanabhan, Nicholas Sirica, Rohit P. Prasankumar, Antoinette J. Taylor, Hou-Tong Chen

**Affiliations:** 1 Center for Integrated Nanotechnologies, Los Alamos National Laboratory, Los Alamos, NM 87545 USA; 2 Deep Science Fund, Intellectual Ventures, Bellevue, WA 98005 USA

**Keywords:** Terahertz optics, Nonlinear optics, Nanophotonics and plasmonics, Ultrafast photonics, Metamaterials

## Abstract

Nonlinear optical spectroscopies are powerful tools for investigating both static material properties and light-induced dynamics. Terahertz (THz) emission spectroscopy has emerged in the past several decades as a versatile method for directly tracking the ultrafast evolution of physical properties, quasiparticle distributions, and order parameters within bulk materials and nanoscale interfaces. Ultrafast optically-induced THz radiation is often analyzed mechanistically in terms of relative contributions from nonlinear polarization, magnetization, and various transient free charge currents. While this offers material-specific insights, more fundamental symmetry considerations enable the generalization of measured nonlinear tensors to much broader classes of systems. We thus frame the present discussion in terms of underlying broken symmetries, which enable THz emission by defining a system directionality in space and/or time, as well as more detailed point group symmetries that determine the nonlinear response tensors. Within this framework, we survey a selection of recent studies that utilize THz emission spectroscopy to uncover basic properties and complex behaviors of emerging materials, including strongly correlated, magnetic, multiferroic, and topological systems. We then turn to low-dimensional systems to explore the role of designer nanoscale structuring and corresponding symmetries that enable or enhance THz emission. This serves as a promising route for probing nanoscale physics and ultrafast light-matter interactions, as well as facilitating advances in integrated THz systems. Furthermore, the interplay between intrinsic and extrinsic material symmetries, in addition to hybrid structuring, may stimulate the discovery of exotic properties and phenomena beyond existing material paradigms.

## Abstract (web summary)

Insights from recent THz emission studies involving quantum materials and low-dimensional systems are surveyed from the standpoints of dynamical mechanisms and basic symmetries, highlighting interplays between intrinsic and designer structuring.

## Graphical abstract

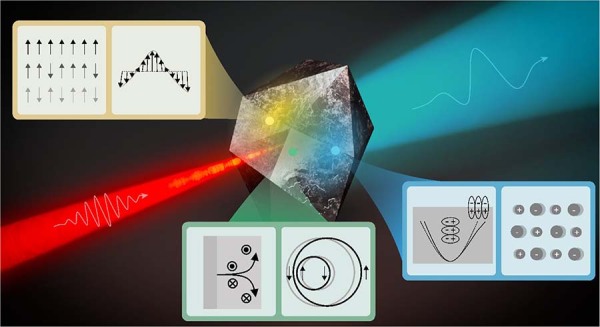

## Introduction

Much of our understanding in physics derives from the study of symmetry and how it underlies the various conservation laws found in nature. Condensed matter systems are naturally understood by their symmetries, and it is through the breaking of these symmetries that many technologically relevant properties emerge, such as magnetism, ferroelectricity, and superconductivity. Conventional probes of symmetry include x-ray, neutron, and electron scattering techniques to determine the lattice, magnetic, and charge ordering in a crystal. Nonlinear optics has also proven to be an effective probe of magnetic point group symmetries^1^, as expressed through nonlinear response functions^2,3^. Nonlinearity in this context refers to a second- or higher-order material response to applied electromagnetic fields. Constraints placed on the response function due to interactions between multiple fields make such techniques especially amenable to revealing novel phases that are otherwise hidden from linear probes^4–7^.

As compared with the more widely utilized second harmonic generation spectroscopy, THz emission (1 THz $=$ 10^12^ s^−1^) provides a complementary method for determining material point groups. As second-order processes, both techniques are highly sensitive to a breaking of local symmetry in the electronic state^8^. This symmetry breaking can occur spontaneously for a continuous phase transition, such as electric polarization across a ferroelectric transition^9^, or explicitly through application of an external electric field^10^ or current pulse^11^. However, as compared to second harmonic generation, THz emission is generally more sensitive to chiral symmetry, since it is not constrained by the permutation symmetry in the same manner as the second harmonic response tensor^12^. Beyond the sensitivity to static point group symmetries, THz emission also has much broader implications for the study of dynamics. In particular, the transduction of optical pulses into electrical signals through photocurrent generation can provide a detailed view of ultrafast (femto–picosecond) energy/momentum flows, changing order parameters, and quasiparticle interactions, while also revealing more fundamental aspects of the electronic structure under highly nonequilibrium conditions^13^. Capturing this time evolution allows for contact-free observation of the microscopic processes contributing to these dynamics^14^ through the emission of THz radiation, which is detected directly though electro-optic sampling^15^, photoconductive antennas^16^, or THz field-induced second harmonic generation^17^.

Optically driven THz emission spectroscopy thus provides access to photocurrents and other dynamics that are not readily observed with other widely utilized THz spectroscopic approaches. These include THz time-domain spectroscopy for measuring the complex THz conductivity and dielectric function^18^, optical–pump THz–probe spectroscopy for measuring the time evolution of the THz conductivity following optical excitation^19^, and two-dimensional coherent THz spectroscopy for observing the kinetics/dynamics of quasiparticle (e.g., phonon or magnon) population, coupling, and coherence^20,21^. Many THz emission studies have indeed been geared toward demonstrating new, efficient sources of intense and broadband THz radiation to facilitate these THz spectroscopies. While some prominent discoveries and opportunities toward this end will be noted, here we emphasize the value of THz emission as a spectroscopic tool in itself.

Following an overview of common THz emission mechanisms, we discuss these light-matter interactions in the more general underlying framework of conserved and broken symmetries. Such considerations will provide the foundation for understanding recent THz emission studies of quantum materials, including strongly correlated, topological, and magnetic systems. The detailed interplay of intrinsic atomic lattice symmetries and extrinsic structural (especially interfacial or micro–nanostructured) symmetries will emerge as an important thematic element among many of these studies, culminating here in an overview of recent work in designer low-dimensional systems. We thereby hope to provide a helpful (if not exhaustive) overview of the essential systems explored thus far via THz emission, offering perspective in the framework of basic symmetries and highlighting opportunities for designing such material and light-matter interaction symmetries in artificially structured systems. Given the breadth of this field, it would be impossible to cover all of the exciting work performed over the past several decades and we apologize to any colleagues whose work we have unintentionally overlooked.

## Symmetries underlying pulsed terahertz emission

**Fig. 1:**
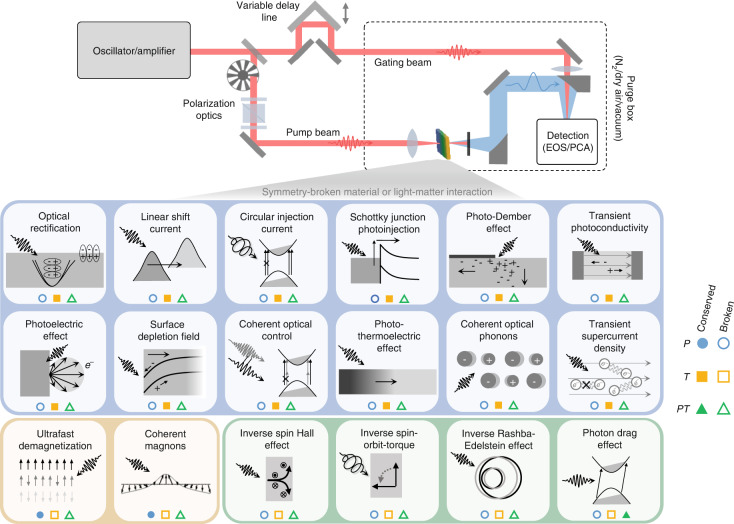
Time-resolved THz emission spectroscopy of symmetry-broken materials. A general THz emission spectroscopy setup is shown in transmission configuration (readily reconfigured into reflection mode), along with a selection of mechanisms by which ultrafast photocurrents and THz radiation are generated. Mechanisms are grouped by the essential broken discrete symmetry, with the symmetry broken either within the material or at an interface, within the light-matter interaction, or via an applied static field. In all cases where only parity (${\mathscr{P}}$) or time-reversal (${\mathscr{T}}$) is broken, parity-time (${\mathscr{P}}{\mathscr{T}}$) symmetry is broken generically

Ultrafast pulses of THz radiation are generated through a variety of mechanisms (Fig. 1), including nonlinear optical rectification, picosecond transient currents, and ultrafast magnetization dynamics. Considering only radiative transverse currents ($\nabla \cdot {\bf{j}}=0$), one finds the general expression for the field radiated to free space^22^^,^^23^,

1 $${{\bf{E}}}_{{\rm{rad}}}\left({\bf{r}},t\right)=-\frac{\partial {\bf{A}}}{\partial t}=-\frac{1}{4\pi {\epsilon }_{0}{c}^{2}}\int \frac{1}{\left|{\bf{r}}-{{\bf{r}}}^{{\prime} }\right|}\frac{\partial {\bf{j}}\left({{\bf{r}}}^{{\prime} },{t}^{{\prime} }\right)}{\partial t}d{{\bf{r}}}^{{\prime} }$$

in which ${\bf{A}}$ is the electromagnetic vector potential, ${\epsilon }_{0}$ is the vacuum permittivity, and ${t}^{{\prime} }=t-\tfrac{|{\bf{r}}\,-\,{{\bf{r}}}^{{\prime} }|}{c}$ is the retarded time between the source (${\bf{r}}^{\prime}$) and measurement (${\bf{r}}$) locations for a radiation field traveling at the speed of light in vacuum ($c$). The total current density,

2 $${\bf{j}}={{\bf{j}}}_{{\rm{f}}}+{{\bf{j}}}_{{\rm{b}}}={{\bf{j}}}_{{\rm{f}}}+\frac{\partial {\bf{P}}}{\partial t}+\nabla \times {\bf{M}}$$

includes free (${{\bf{j}}}_{{\rm{f}}}$) and bound (${{\bf{j}}}_{{\rm{b}}}$) current contributions. The free current density involves a variety of (often competing) processes described below, while the bound current involves both linear and nonlinear polarization (${\bf{P}}$) and magnetization (${\bf{M}}$) orders. Further expansion of Eq. (1) in terms of electric and magnetic multipoles^22^ shows that Eqs. (1) and (2) generically encode all mechanisms of pulsed THz emission, the most common of which involve second-order nonlinear processes where the THz field grows linearly with the incident optical intensity, ${E}_{{\rm{THz}},i}\propto {E}_{j}{E}_{k}$. The constitutive relations for different mechanisms may then be determined in terms of complex susceptibilities (${\chi }_{{ijk}}^{\left(2\right)}$), conductivities (${\sigma }_{{ijk}}^{\left(2\right)}$), or other tensors, as we now briefly examine for each term in Eq. (2).

While many processes involving the down-conversion of optical to THz photons are commonly referred to as optical rectification, for clarity we reserve this term for the coherent nonlinear polarization contribution, ${P}_{i}^{\left(2\right)}\left(\Omega \right)={\epsilon }_{0}{\chi }_{{ijk}}^{\left(2\right)}\left(\Omega \approx 0;{\omega }_{1},\,-{\omega }_{2}\right){E}_{j}\left({\omega }_{1}\right){E}_{k}^{* }({\omega }_{2})$, with a summation implied on repeated spatial indices $j$ and $k$. Then ${{\bf{E}}}_{{\rm{THz}}}\left(t\right)\propto -\tfrac{\partial {{\bf{j}}}_{{\rm{b}},{\rm{P}}}}{\partial t}=-\tfrac{{\partial }^{2}{{\bf{P}}}^{\left(2\right)}}{\partial {t}^{2}}$, in which ${{\bf{j}}}_{{\rm{b}},{\rm{P}}}$ is the transient bound (virtual carrier) current density generated by the time-varying nonlinear polarization, ${{\bf{P}}}^{(2)}(t)$ (understood to be the inverse Fourier transform of ${{\bf{P}}}^{(2)}(\Omega )$, for notational simplicity). This rectified polarization field follows the pulse intensity envelope in the time domain, which is observed in the frequency domain as difference frequency generation between optical frequency components spanning the pulse bandwidth ($\sim$4.4 THz for a 100 fs bandwidth-limited Gaussian optical pulse). For noncentrosymmetric semiconductors with nonvanishing ${\chi }_{{ijk}}^{(2)}$ elements, optical rectification is the prevailing contribution to THz emission in the case of below-bandgap excitation^24^. Examples include zincblende crystals (e.g., ZnTe, GaP, and GaAs), wurtzite crystals (e.g., CdS and CdSe), and other members of the 21 noncentrosymmetric crystallographic point groups. Phase matching between the phase velocity of the THz field and the group velocity of the optical pulse must also be considered in this bulk rectification process^15^. Surface rectification can additionally occur at the interfaces between media with bulk centrosymmetry, complementing surface-localized second harmonic generation^25^ in studies of interfacial structure, fields, and chemical composition (sensing).

A rich variety of processes driven by the above-bandgap excitation of ultrafast free-carrier photocurrents can also be investigated through the emitted THz waveforms, with ${{\bf{E}}}_{{\rm{THz}}}\left(t\right)=-\tfrac{\partial {{\bf{j}}}_{{\rm{f}}}}{\partial t}$. The most common contributions to this free current density are drift and diffusion processes. Drift currents are generated by charge acceleration within applied or built-in electric fields, as occur within semiconductor surface depletion regions^26^, p–n junctions, or Schottky junctions. Due to the exposed interface, currents driven by surface depletion fields are particularly sensitive to external influences such as oxide formation and adsorbed molecular layers^27–29^. Diffusion currents, on the other hand, are driven by localized excitations and uneven carrier mobilities in photo-Dember^30^ or photo-thermoelectric^31^ effects. In longitudinal photo-Dember THz emitters such as InAs crystals^32,33^, excited electrons near the crystal surface diffuse more quickly into the bulk than the corresponding holes, creating currents in the direction normal to the surface (i.e., out-of-plane electric dipole). The outcoupling efficiency of THz radiation along the specular direction is constrained by the dipolar radiation pattern and the escape cone arising from total internal reflection^34^. To facilitate THz outcoupling while also introducing the ability to control the polarization with sample orientation, lateral (in-plane) photo-Dember THz emission has also been demonstrated in semiconductors^35^, as well as in graphene^36^, by exploiting partial shading of the surface, e.g., with micro-patterned gold stripes. This leads to asymmetric spatially varying lateral carrier densities and corresponding directional in-plane THz currents. Lateral current contributions and enhanced outcoupling have also been achieved by application of a magnetic field, leading to rotation of the surge current THz dipole under the Lorentz force^34,37,38^.

Two other important contributions to ${{\bf{j}}}_{{\rm{f}}}$ are the shift and injection currents, together making up the so-called bulk photovoltaic effect^39,40^, which represents a new pathway for solar energy conversion beyond traditional p–n junction photovoltaics^41^. We note that labels such as photovoltaic and photogalvanic—the relative definitions of which have yet to be consistently established—are largely omitted in the present discussion in favor of more physically transparent terms, though such labels are utilized in line with the relevant literature where they do appear. Shift currents are generated during photoexcitation between initial and final states with different centers of charge, leading to coherent shifting of the real-space charge density. Injection currents, on the other hand, involve asymmetric carrier excitation in $k$-space with a net group velocity, due to helicity-dependent quantum interference between different polarization components of a circularly polarized excitation beam^42,43^. These currents are described by the relations,

3 $${j}_{{\rm{shift}},i}\left(\Omega \right)={\sigma }_{{ijk}}{E}_{j}\left({\omega }_{1}\right){E}_{k}^{* }({\omega }_{2})$$

4 $$\frac{\partial {j}_{{\rm{inj}},i}\left(\Omega \right)}{\partial t}={\eta }_{{ijk}}{E}_{j}{\left({\omega }_{1}\right)E}_{k}^{* }({\omega }_{2})$$

where ${\sigma }_{{ijk}}$ and $\tfrac{i{\eta }_{{ijk}}}{\omega }$ are, in general, complex conductivity tensors, with the factor of $\tfrac{i}{\omega }$ in the latter due to the time derivative in Eq. (4). In most systems studied so far, ${\sigma }_{{ijk}}\left(0;\omega ,-\omega \right)={\sigma }_{{ikj}}\left(0;\omega ,-\omega \right)$ is purely real and corresponds to the linear shift current, while ${\eta }_{{ijk}}\left(0;\omega ,-\omega \right)={\eta }_{{ikj}}\left(0;-\omega ,\omega \right)=-{\eta }_{{ikj}}\left(0;\omega ,-\omega \right)={\eta }_{{ijk}}^{* }(0;-\omega ,\omega )$ is purely imaginary and corresponds to the circular injection current. However, it is now understood that circular shift currents and linear injection currents can also occur in magnetic systems with broken time-reversal symmetry^40,44^. Furthermore, although shift and injection currents have only been observed in noncentrosymmetric media or at the surfaces of centrosymmetric media^45^, recent theory suggests that even this basic requirement of inversion symmetry breaking may be circumvented in the case of photon-drag-mediated non-vertical excitations^46,47^.

A nonzero photon drag current can occur even in centrosymmetric crystals due to the transfer of photon momentum during absorption. The momentum of an individual photon is small in the optical regime yet can lead to appreciable currents using intense optical beams with high photon flux^48,49^. This process is described by a fourth-rank tensor, which emerges upon expanding the conductivity to first order in the photon momentum^50,51^, ${\bf{q}}$, as

5 $${j}_{{\rm{drag}},i}\left(\Omega \right)={T}_{{ijkn}}{q}_{n}{E}_{j}{\left({\omega }_{1}\right)E}_{k}^{* }({\omega }_{2})$$

Finally, ultrafast magnetization dynamics can also yield THz radiation, ${E}_{{\rm{THz}}}\left(t\right)\propto -\tfrac{\partial {{\bf{j}}}_{{\rm{b}},{\rm{M}}}}{\partial t}=-\tfrac{\partial \left(\nabla \times {\bf{M}}\right)}{\partial t}$, in which ${{\bf{j}}}_{{\rm{b}},{\rm{M}}}=\nabla \times {\bf{M}}$ is the transient magnetization-induced bound current density from Eq. (2). In the case of ultrafast demagnetization, this is often expressed explicitly as^52^ ${E}_{{\rm{THz}},y}\left(t\right)=\tfrac{{\mu }_{0}}{4{\pi }^{2}}\int \tfrac{1}{|{\bf{r}}-{{\bf{r}}}^{{\prime} }|}\tfrac{{\partial }^{2}{M}_{x}({{\bf{r}}}^{{\prime} },{t}^{{\prime} })}{\partial {t}^{2}}d{{\bf{r}}}^{{\prime} }$. Here, the term “bound” refers to the spin polarization rather than free charge motion (i.e., current loops), with contributions from itinerant electrons as well as site-localized spins. However, bound magnetization currents are not the only magnetic contributions to THz transient currents, as free charge currents (${{\bf{j}}}_{{\rm{f}},{\rm{M}}}$) are also generated when pure spin currents are converted to charge currents. This can occur in a variety of inverse effects at interfaces between magnetic and non-magnetic materials, as with the inverse spin Hall^53^ and inverse Rashba-Edelstein^54,55^ effects.

The mechanistic viewpoint described above offers system-specific details on physical properties, dynamics, and relative current contributions. Yet, THz emission also reveals the underlying symmetries of a system, which determine the allowed processes via vanishing or nonvanishing tensor elements. As the order of nonlinearity and the rank of the corresponding tensors increases, so does the available information on crystal structure, dynamical couplings, and symmetries. At the most basic level, optically driven THz emission requires an explicit or spontaneous directionality within the material system or light-matter interaction, which requires the breaking of either parity/inversion (${\mathscr{P}}$), time-reversal (${\mathscr{T}}$), or combined ${\mathscr{P}}{\mathscr{T}}$ symmetry. Some aspects of these discrete symmetries are introduced below, with a selection of corresponding mechanisms and their broken symmetries summarized in Fig. 1. The bulk of this Review is then devoted to exploring recent insights into the physical properties, dynamical mechanisms, and broken symmetries underlying ultrafast THz emission from emerging material systems.

### ${\mathscr{P}}$ Symmetry breaking

A system exhibiting parity symmetry remains unchanged to within an overall phase factor under spatial inversion, $\left(x,y,z\right)\to (-x,-y,-z)$. The parity operator is Hermitian, ${\mathscr{P}}{\mathscr{=}}{{\mathscr{P}}}^{\dagger }$, and unitary, ${{\mathscr{P}}}^{\dagger }={{\mathscr{P}}}^{-1}$, with ${{\mathscr{P}}}^{2}=1$. In systems with inversion symmetry, ${\mathscr{P}}$ commutes with the Hamiltonian, $\left[{\mathscr{P}}{\mathscr{,}}{\mathscr{H}}\right]=0$, such that the energy eigenstates are also eigenstates of the parity operator, ${\mathscr{P}}\left|\psi \left({\bf{r}},t\right)\right\rangle =\left|\psi \left({\boldsymbol{-}}{\bf{r}},t\right)\right\rangle =\pm \left|\psi \left({\bf{r}},t\right)\right\rangle$. Polar vectors such as position, linear momentum, and electric field are odd under parity, while pseudovectors such as angular momentum (spin and orbital) and magnetic field are even.

Net polarization or photocurrent generation requires a defined system directionality and may thus occur in systems with broken ${\mathscr{P}}$ symmetry ($\left[{\mathscr{P}}{\mathscr{,}}{\mathscr{H}}\right]\ne 0$). The Hamiltonian, ${\mathscr{H}}$, must therefore contain at least one ${\mathscr{P}}$-odd term. A standard example is the anharmonic oscillator potential, $V\left(x\right)=\tfrac{1}{2}m\omega {x}^{2}+a{x}^{3}$, expanded locally about $x=0$, where the second term is a small perturbation and ${{\mathscr{P}}}^{\dagger }{x}^{3}{\mathscr{P}}{\mathscr{=}}{\mathscr{-}}{x}^{3}$. This model can be utilized as an approximation for the unit cell potential in noncentrosymmetric crystals^56^, or more generally for interfacial potentials.

While the breaking of ${\mathscr{P}}$ symmetry is necessary for many THz emission mechanisms, it is insufficient to determine which (if any) components of ${\chi }_{{ijk}}^{\left(2\right)}$ or other tensors will be nonvanishing. Circular injection currents, for instance, can occur within 18 of the 21 noncentrosymmetric crystal classes, but are forbidden for $\bar{4}3m$ (zincblende), $\bar{6}m2$, and $\bar{6}$ crystals, due to the antisymmetry of ${\eta }_{{ijk}}$ under permutation of the last two coordinates^42^. A full group theoretic analysis^1^ is therefore often necessary to extract the greatest insight on the crystal symmetries, quasiparticle interactions, and currents underlying the various THz emission mechanisms.

The ${\mathscr{P}}$ symmetry breaking at interfaces plays a key role in a variety of THz dynamics, so it is unsurprising that many new properties emerge in low-dimensional systems such as 2D wells, 1D wires, and nanostructures. As we shall describe below, recent studies on nanostructured systems demonstrate how artificial spatial symmetries can introduce or enhance otherwise forbidden or weak THz dipoles, offering new insights into deeply sub-THz-wavelength and even sub-optical-wavelength physics.

### ${\mathscr{T}}$ Symmetry breaking

A system exhibiting ${\mathscr{T}}$ symmetry remains unchanged to within an overall phase factor under time reversal, $t\to -t$, with $\left[{\mathscr{T}}{\mathscr{,}}{\mathscr{H}}\right]=0$. Unlike parity, the time reversal operator must be *anti*unitary, such that ${\mathscr{T}}i=-i{\mathscr{T}}$, and may thus be generally written as the product of a system-dependent unitary operator (${\mathscr{U}}$) and the complex conjugation operator (${\mathscr{C}}$), ${\mathscr{T}}{\mathscr{=}}{\mathscr{U}}{\mathscr{C}}$. Both linear and angular momentum are odd under ${\mathscr{T}}$, as are the vector potential and magnetic field (${\bf{B}}=\nabla \times {\bf{A}}$), while the position and electric field are even (${\bf{E}}=-\tfrac{\partial {\bf{A}}}{\partial t}$, for zero scalar potential).

Time-reversal symmetry breaking ($\left[{\mathscr{T}}{\mathscr{,}}{\mathscr{H}}\right]\ne 0$) underlies a variety of THz emission mechanisms in magnetic systems. Showing this explicitly at the Hamiltonian level can be subtle, however, as many terms involve vector products such as ${\bf{v}}\times {\bf{B}}$ (where ${\bf{v}}$ is the velocity of a charged particle) or ${\bf{B}}\cdot {\bf{S}}$ (where ${\bf{S}}$ is some spin angular momentum). In such terms, both components are evidently ${\mathscr{T}}$-odd (e.g., ${\mathscr{T}}{\bf{B}}{{\mathscr{T}}}^{-1}=-{\bf{B}}$ and ${\mathscr{T}}{\bf{S}}{{\mathscr{T}}}^{-1}=-{\bf{S}}$), leading to an overall ${\mathscr{T}}$-even term in the Hamiltonian, ${\mathscr{T}}{\mathscr{(}}{\bf{B}}{\boldsymbol{\cdot }}{\bf{S}}{\boldsymbol{)}}{{\mathscr{T}}}^{-1}=({\bf{B}}{\boldsymbol{\cdot }}{\bf{S}}{\boldsymbol{)}}$. This apparent contradiction with the known ${\mathscr{T}}$ symmetry breaking in the case of a Hall effect in an applied magnetic field^57^, for instance, can be resolved with careful separation of the ${\mathscr{T}}$-broken subsystem and ${\mathscr{T}}$-invariant “external” system. The system generating this external magnetic field may be treated as invariant under ${\mathscr{T}}$, such that ${\mathscr{T}}{{\bf{B}}}_{{\rm{ext}}}{{\mathscr{T}}}^{-1}={{\bf{B}}}_{{\rm{ext}}}$ and ${\mathscr{T}}\left({{\bf{B}}}_{{\rm{ext}}}{\boldsymbol{\cdot }}{\bf{S}}\right){{\mathscr{T}}}^{-1}=-({{\bf{B}}}_{{\rm{ext}}}{\boldsymbol{\cdot }}{\bf{S}}{\boldsymbol{)}}$. In the case of spontaneous magnetic ordering, as in a ferromagnet below the Curie temperature, the orientation is randomly selected by the spontaneous symmetry breaking and therefore not subject to the direction of time. The resulting magnetization may be separated out as an external mean field, ${{\bf{M}}}_{{\rm{ext}}}$, which remains invariant under application of ${\mathscr{T}}$ to the remaining subsystem. In both cases the ${\mathscr{T}}$ breaking of the relevant subsystem Hamiltonian becomes explicit, $\left[{\mathscr{T}}{\mathscr{,}}{{\mathscr{H}}}_{{\rm{sub}}}\right]\,\ne \,0$. Optically-induced THz demagnetization, by contrast, breaks ${\mathscr{T}}$ via dissipation, which is non-Hermitian and thus generally less amenable to a Hamiltonian description, though it can be described in terms of energy flow from the subsystem into an environment/bath.

### ${\mathscr{P}}{\mathscr{T}}$ Symmetry breaking

If either ${\mathscr{P}}$ or ${\mathscr{T}}$ symmetry is broken while the other is conserved, ${\mathscr{P}}{\mathscr{T}}$ symmetry is broken generically and thus offers no additional insight. Many systems also exist in which broken ${\mathscr{P}}$ and ${\mathscr{T}}$ symmetries lead to independent processes for which combined ${\mathscr{P}}{\mathscr{T}}$ breaking is not essential, as with simultaneous surface optical rectification and optically induced demagnetization^58^. In other systems, overall ${\mathscr{P}}{\mathscr{T}}$ breaking is responsible for photocurrent generation while only ${\mathscr{P}}$ or ${\mathscr{T}}$ may be broken locally in different spatial regions. We will refer to this simply as “separated” ${\mathscr{P}}{\mathscr{T}}$ breaking. This occurs in the inverse spin Hall effect, which involves the combination of spin current generation in a ${\mathscr{T}}$-broken ferromagnetic material that is transformed to a net charge current due to a nonzero net momentum defined by a ${\mathscr{P}}$-broken interface (analogous to a photoemission process) with a non-magnetic material^53,59^, as described further below. There are other systems for which true local ${\mathscr{P}}{\mathscr{T}}$ symmetry breaking is responsible for the charge current response. One instance of this is the inverse spin-orbit torque effect, which occurs directly at interfaces of magnetic heterostructures^54,55^. The interfacial ${\mathscr{P}}$ symmetry breaking acts with an optically-induced effective magnetic field (breaking ${\mathscr{T}}$) to drive directional charge currents localized at the interface.

Radiative ultrafast currents can also occur when ${\mathscr{P}}{\mathscr{T}}$ symmetry is conserved but both ${\mathscr{P}}$ and ${\mathscr{T}}$ are broken, as in some centrosymmetric antiferromagnets^44^. It has been shown recently that circular shift and linear injection currents can arise in such ${\mathscr{P}}{\mathscr{T}}$-symmetric systems, with an underlying description in terms of quantum geometry^44^. The most general symmetry analysis related to spatial arrangements of charge and spins, and corresponding insights from THz emission studies, can be performed in terms of magnetic point groups^1^. It is interesting to note that ${\mathscr{P}}{\mathscr{T}}$-conserved (but ${\mathscr{P}}$ and ${\mathscr{T}}$ broken) second-order nonlinear responses can also emerge in the presence of net linear momentum, such as in the photon drag effect (with net photon momentum) or transient photocurrents (net charge momentum)^11,60^. A transient-photocurrent-induced nonlinearity is a cascaded ${\chi }^{(2)}+{\chi }^{(2)}$ process, with the existing transient current serving as the primary source of THz radiation and the induced nonlinearity read out via second harmonic generation, although a weak induced THz rectification will also occur.

## Transient currents in strongly correlated and topological materials

The strength of THz emission spectroscopy stems from its sensitivity to symmetry breaking in the electronic state. Nowhere is this more apparent than in strongly correlated and topological material systems, as the presence of frustrated couplings provides an opportunity to explore unconventional symmetries and symmetry breaking that would otherwise be forbidden based on crystal structure alone. Here we will focus on the breaking of ${\mathscr{P}}$ symmetry brought on intrinsically within some broken symmetry states or extrinsically by application of an external bias field. Within strongly correlated materials, such as high-${T}_{{\rm{C}}}$ superconductors or multiferroic oxides, we will focus on the use of THz emission to gain insights into the ordered ground state, while the nonlinear response in topological materials can go one step further by shedding light on the quantum geometrical properties that are characteristic of the topological state. Notably, light-induced photocurrents will feature prominently in the discussion, where we emphasize that THz emission in general provides an all-optical, contact-free means of tracking photocurrents that largely mitigates extraneous effects due to Schottky barriers, field screening, and generation of interfacial defects common to direct photocurrent readout. However, as compared to the continuous excitation used in static photocurrent measurements, pulsed excitations employed in THz emission can have unintended consequences, including laser heating. Often the degree to which heating influences the emitted THz spectra is material specific and can frequently be identified by a lack of polarization dependence in the spectra. Nonetheless, heating can be responsible for driving either reversible or irreversible phase transitions within a material and should be carefully monitored during the experiment.

### Superconductors

**Fig. 2:**
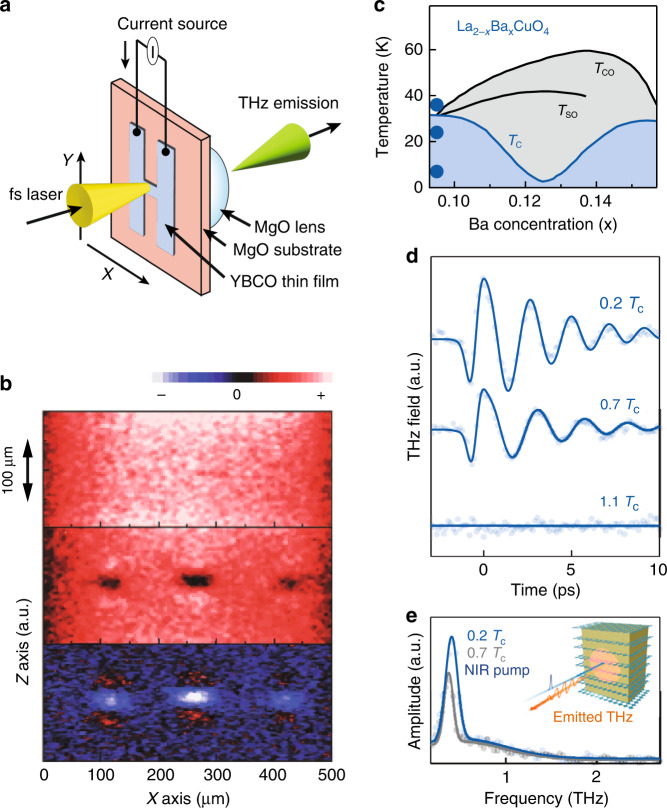
THz emission from high-*T*_**C**_ superconductors. **a** Schematic illustration of a superconducting dipole antenna. **b** Image of superconducting strip line before illumination (top), after illumination (middle), and following removal of the bias current (bottom). Note the formation of flux vortices following illumination, which subsequently revert to vortex/anti-vortex pairs upon removal of the bias. **c** Temperature phase diagram for the incommensurate stripe-ordered compound La_1.905_Ba_0.095_CuO_4_. Here, ${T}_{{\rm{CO}}}$, ${T}_{{\rm{SO}}}$, and ${T}_{{\rm{C}}}$ represent the charge ordering, spin ordering, and the superconducting critical temperatures, respectively. **d** Emitted THz waveforms obtained for temperature intervals denoted by solid circles in (**c**). **e** Spectral amplitude of select time-domain traces in (**d**) fit to multi-Gaussian fit function. Inset: Schematic of the experimental geometry. Panel (**a**) adapted with permission from^63^ © 2019 Wiley-VCH Verlag GmbH. Panel (**b**) adapted with permission from ref. ^66^ © 2005 The Japan Society of Applied Physics. Panels (**c**–**e**) reprinted from ref. ^71^ © 2022 the Author(s), published by PNAS, under the terms of CC-BY 4.0

Current/voltage biased superconductors are known to emit THz radiation, as the formation of a superconducting gap in the quasiparticle excitation spectrum leads to carrier dynamics that closely mimic that of narrow bandgap semiconductors^61,62^. Much like photoconductive switches, THz emission from biased superconductors results from time-dependent modulations of the supercurrent density brought on by the breaking of Cooper pairs following impulsive optical excitation^63,64^ (Fig. 2a). Such emission can lead to persistent nonequilibrium dynamics attributed to avalanche pair breaking, whereupon initial pair breaking triggered by a single absorbed photon drives secondary pair breaking due to multi-scattering of hot carriers during the relaxation process^65^.

In high-${T}_{{\rm{C}}}$ cuprates, which are classified as type-II superconductors capable of supporting vortices of quantized magnetic flux, the partial suppression of supercurrent density following optical excitation can transiently introduce magnetic flux in a manner akin to field cooling. Here, the attenuated field generated by perturbed supercurrents within the illuminated region cannot compensate for that generated by unperturbed supercurrents outside this region^66^. This magnetic flux quantizes as the system relaxes back towards a superconducting ground state, yielding vortex/anti-vortex pair bundles whose distribution reflects the beam profile of the optical excitation pulse^66^. Since the emission of THz radiation from biased superconductors results from such *local* modulation of the supercurrent, imaging of vortex pair bundles can be accomplished by scanning the optical beam across the sample following the removal of a bias current (Fig. 2b). Here, precise patterning of a superconducting strip line facilitates the pinning of these pair bundles, enabling ultrafast control over reading and writing of these topological defects in the superconducting state^63,66^.

Aside from supercurrent modulation, high-${T}_{{\rm{C}}}$ cuprates can likewise emit THz radiation under bias due to tunneling of Cooper pairs through the intrinsic Josephson junctions that develop between superconducting CuO_2_ layers and insulating BiO/SrO barrier layers, as occurs naturally within Bi_2_Sr_2_CaCu_2_O_8+x_ (Bi-2212)^67,68^. While a given crystal can consist of many thousands of these junctions, the challenge comes in achieving coherence among individual emitters. This can be accomplished through application of an external magnetic field to create coherent Josephson vortex flows^69^, or by supporting electromagnetic standing waves, which act as longitudinal cavity modes within the crystal^70^. Here, emission in the far-field results from the coherent superposition of THz radiation from each individual emitter, resulting in a THz field that scales linearly with the number of junctions, while the emission frequency can be continuously tuned through varying the bias voltage across each junction.

Thus far the emission of THz radiation from high-${T}_{{\rm{C}}}$ superconductors has relied on an external bias to break ${\mathscr{P}}$ symmetry, as intrinsic second-order nonlinear processes are symmetry forbidden within these centrosymmetric compounds. However, the presence of frustrated couplings, as found within the stripe-ordered cuprate La_2-x_Ba_x_CuO_4_, offers a new opportunity to explore hidden electronic symmetry, showcasing the utility of THz emission as a spectroscopic probe of quantum materials^71^. Here, the emission of narrow-band THz radiation following optical excitation occurs when fluctuating or incommensurate charge stripes coexist with superconductivity (Fig. 2c–e), leading to a breaking of inversion symmetry between CuO_2_ planes. In this work, emission is argued to arise from surface Josephson plasmons, which cannot generally couple to light fields but can do so here as a result of Umklapp-like scattering off the stripe order^71^.

### Multiferroics

Much of the technological appeal of strongly correlated electron systems stems from the ability to indirectly manipulate charge, spin, and orbital degrees of freedom by exploiting the strong coupling present between these various quantities. Multiferroics, which are characterized by the existence of two or more ferroic orders in the same phase, are prime examples where the coupling between, e.g., ferroelectric and ferromagnetic orders, can be used to electronically manipulate the magnetic state for memory applications^72^. Multiferroics fall into two classes:^73^ type I, in which the noncentrosymmetric lattice distortion responsible for driving ferroelectric order occurs independent of magnetic order, and type II, where ferroelectricity is induced by spinoidal magnetic order. While the former possesses weaker coupling between ferroelectric and ferromagnetic orders, the fact that it develops a net electric polarization at or above room temperature has led to extensive investigations using THz emission spectroscopy.

As a prototypical type I multiferroic with a Curie temperature >1000 K, BiFeO_3_ (BFO) can produce THz emission from a variety of physical processes, including optical rectification^74^ and photocurrent generation^75^. Here, the dominant mechanism largely depends on photon energy relative to the 2.6 eV direct bandgap, with optical rectification dominating for below bandgap excitation, while photocurrent generation dominates above. In both cases, contributions from ultrafast modulation of the electric polarization (${E}_{{\rm{THz}}}\propto \,-\,\tfrac{{\partial }^{2}P}{\partial {t}^{2}}$) plays a key role. The ferroelectric axis of BFO can be along any of the four long diagonals of the pseudo-cubic unit cell, leading to eight possible ferroelectric domains in the crystal. As expected for a nonlinear probe, crystallographic orientation and lattice strain^76^ can significantly affect the efficiency of THz emission, as fundamentally different behavior can be seen depending on whether the ferroelectric polarization is contained within the crystallographic plane^77^. Furthermore, photocurrent generation arising from mono- or stripe-ordered domains following above-bandgap excitation can likewise have distinct origins^78^. Here, it was found that the net photocurrent in the stripe-ordered phase is dominated by charge separation across the domain walls, while monodomain samples exhibit bulk shift currents associated with the noncentrosymmetry (broken ${\mathscr{P}}$) of the crystal. The peak current amplitude driven by the charge separation at domain walls is found to be two orders of magnitude higher than the bulk shift current response, indicating the prominent role that domain walls play as nanoscale junctions to efficiently separate photogenerated charges in BFO.

### Topological insulators and semimetals

While the discussion of strongly correlated electron systems has focused on the use of THz emission to gain insight into the symmetry-broken ground state, the discovery of novel topological phases—characterized by topological invariants as opposed to some local order parameter—provides an alternative framework for classifying states of matter^79,80^. Nevertheless, symmetry continues to play a central role in the physics of topological materials, as it underlies topological protection in topological insulators and superconductors^81^, crystalline topological phases^82^, and the recently discovered topological semimetals^83,84^.

Nonlinear optical probes are well suited to investigate the underlying point group symmetries that protect topological invariants, but recent attention on the role of quantum geometry and topology in the nonlinear electromagnetic response has taken center stage, particularly within the topological semimetals^12,85^. Here, local geometric properties can be distinguished from global topological properties by considering the behavior of an electronic wave function about either an isolated point or closed path in the Brillouin zone. Shift currents, resulting from the real space coherent shift of electron density following photoexcitation^42^, can be traced to a local geometric property defined by the difference in Berry connection between bands participating in the optical transition^86,87^. This has been widely studied in topological insulators^88,89^ and Weyl semimetals^90,91^, where polarization-dependent photocurrent measurements in the latter point towards a geometric contribution to the shift current, revealed by a colossal bulk photovoltaic effect attributed to divergent Berry curvature near the Weyl nodes. This has sparked interest in the use of topological semimetals as broadband photodetectors, where the issue of intrinsically high dark currents common to topologically trivial, gapless semimetals can be largely circumvented by exploiting these geometric aspects of the nonlinear optical response^92^.

**Fig. 3:**
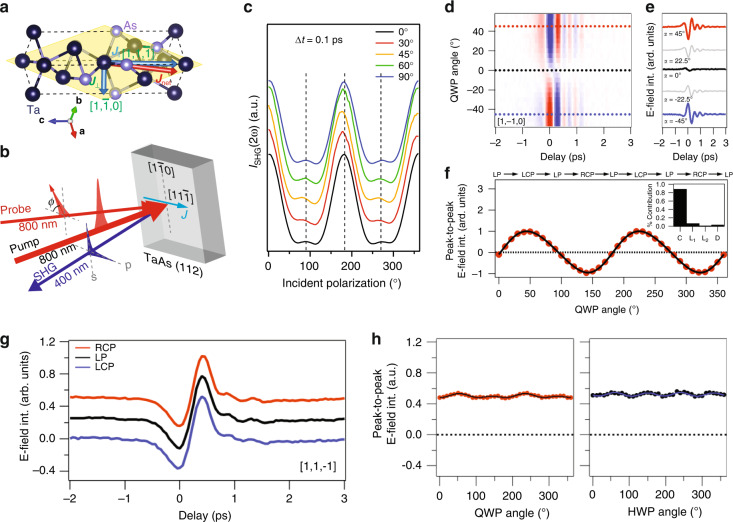
Transient photocurrent-induced symmetry breaking in the Weyl semimetal TaAs. **a** Schematic diagram of the net photocurrent contained within the (112) plane (yellow) of a TaAs unit cell, along with (**b**) a sketch of the experimental geometry used to realize current-induced second harmonic generation. **c** Changes in the transient second harmonic pattern ($\Delta t=$ 0.1 ps) measured as a function of pump polarization relative to the [$1$,$1$,$\bar{1}$] axis (offset for clarity). The presence of enhanced (10–20%), polarization-dependent injection (panels **d**–**f**) and shift (panels **g**, **h**) currents along [$1$,$\bar{1}$,0] leads to a clear reduction in symmetry within the pattern. **d** False color plot and (**e**) select time-domain THz traces, illustrating the polarity reversal of the emitted THz waveform generated along the [$1$,$\bar{1}$,0] axis by injection photocurrents. Traces shown in panel **e** are obtained using quarter waveplate (QWP) angles of $\pm$45°, $\pm$22.5°, and 0°, which correspond to right/left circular, elliptical, and linear polarizations, respectively. **f** Peak-to-peak electric field amplitude plotted as a function of QWP angle. **g** Polarization independent shift currents generated along the [$1$,$1$,$\bar{1}$] axis following excitation by right-circular, linear, and left-circular polarized optical pulses. Detailed peak-to-peak E-field amplitude of the emitted THz radiation plotted as a function of (**h**) QWP (left) half waveplate (HWP; right) angle. Panels (**a**–**c**) adapted with permission from^60^ © 2021 the Author(s), under exclusive licence to Springer Nature. Panels (**d**–**h**) reprinted with permission from ref. ^51^ © 2019 American Physical Society

Shift current generation can be studied in the time domain by THz emission spectroscopy, where added dynamical insights, including that of ligand charge transfer in three-dimensional topological insulators^93^, can be gained by measuring the bandwidth of the emitted THz pulse^51^. By manipulating such an optically driven photocurrent on the ultrafast timescales intrinsic to its generation and decay, it is possible to generically break electronic symmetries through exploiting the polarization dependence of photocurrents underlying the linear photogalvanic effect^60^. This has important implications for topological semimetals, where symmetry is intimately tied to topology, as a local change in the spatial distribution of the electronic wave function brought on by shift current excitation can serve to non-locally influence the electronic structure over the whole momentum space. Considering that the photocurrent itself is expected to transiently break all magnetic point group symmetries, including time reversal, a resultant current-induced second harmonic response can be generated away from high symmetry axes of the crystal, as it is not constrained by crystallographic symmetry in the same way as static harmonic generation^11^. As this pertains to the transition metal monopnictide family of Weyl semimetals (Fig. 3), the subsequent recovery of equilibrium symmetry following shift current excitation reflects time-dependent changes to the polarization distribution, whose relaxation is governed by a momentum-dependent recovery, describing the return in skew to the electronic polarization back to its equilibrium value^94^.

While it is possible to frame the nonlinear optical response of topological materials in terms of local geometric quantities originating from interband Berry connection^44,95^, obtaining an unambiguous measure of global topology is considerably more challenging^96^. To do so, injection currents, arising from the asymmetric distribution of photoexcited carriers in momentum space, have been argued to capture the effects of Berry curvature in topological insulators^97,98^, as well as Weyl fermion chirality in topological semimetals^99,100^. However, the experimental signature of topology is quantization, and being able to measure quantization in the injection photocurrent requires optically allowed transitions to enclose a single topologically protected crossing^101^. Within the Weyl semimetals, this can only occur if nodes of opposite chirality are separated in energy, requiring an absence of mirror symmetry, as can be found in the class of chiral semimetals known as multifold compounds^102–104^. As compared to chiral currents arising from the anomaly, quantization of the injection current is not a topologically protected quantity, as it depends on non-universal parameters such as the scattering rate^101^. In practice, this means that quantization can be readily degraded by perturbative contributions from disorder and electron interactions^105^, but as material quality improves, so does the prospect of obtaining such a clear signature of global topology in the nonlinear response.

As a final note, the fact that topological insulators are centrosymmetric means that inversion symmetry breaking from topologically protected surface or edge states can dominate the nonlinear response. This is generally not the case for Weyl semimetals, as the breaking of inversion symmetry within the crystal naturally ensures that bulk states contribute most strongly to nonlinear effects. However, the bulk-boundary correspondence guarantees a surface manifestation of topology in the Weyl semimetals, which is given by the termination of open energy contours at the surface projection of Weyl nodes, referred to as Fermi arcs^106^. Recent theoretical predictions have suggested that photocurrents resulting from Fermi arc surface states can be separated from bulk injection currents arising within nonsymmorphic crystal structures^107^. This has been experimentally verified in the multifold compound RhSi, where surface shift and injection currents have been distinguished from their bulk counterparts on the basis of symmetry^108^. Such work highlights the ability of nonlinear optical probes like THz emission to gain new insights into both symmetry and quantum geometrical properties of topological materials, even if such insights are unexpected, as occurred with the observation of an emergent mirror symmetry at the surface of RhSi that is inconsistent with its magnetic point group.

## THz emission from transient spin dynamics

The connection between the emission of THz radiation and magnetic materials has its origins in the seminal discovery that ultrafast laser pulses can drive the femtosecond demagnetization of metallic ferromagnetic (FM) films^109^. This is due to energy and angular momentum transfer between the electronic, lattice, and spin degrees of freedom in the material, a complex collection of processes that are typically modeled phenomenologically^109,110^. Ultrafast demagnetization has now been explored in numerous material classes, including magnetic semiconductors^111,112^, dielectrics^113,114^, half-metallic systems^115–117^, and low-dimensional magnetic crystals^118,119^. Given that the ultrafast pump pulse drives a sudden time-varying magnetization in the crystal, classical Maxwell theory predicts the emission of radiation in the far field as ${E}_{x,y}\propto \tfrac{{\partial }^{2}{M}_{y,x}}{\partial {t}^{2}},$ where $E$ is the emitted electric field and $M$ is the magnetization of the material. In 2004, Beaurepaire and coworkers experimentally observed this phenomenon by simultaneously measuring the rapid demagnetization and concomitant emission of THz radiation from an FM nickel film^52^, related to the breaking of ${\mathscr{T}}$ symmetry in the presence of dissipation. In the last two decades, THz emission has been observed from numerous other FM crystals, amorphous magnetic alloys^120^, and heterostructures with magnetic constituents^121^. Indeed, THz emission spectroscopy has emerged as a potentially valuable contact-free probe of magnetization dynamics in a variety of materials^122^.

Nevertheless, despite its seeming ubiquity, THz generation originating purely from ultrafast demagnetization has seen limited technological utility. This is due to the lack of tunability of the THz response, stemming from the fact that the underlying demagnetization dynamics are typically governed by intrinsic and fixed material properties. Parameters such as emission bandwidth, polarization state, and field intensity are therefore inextricably tied to the choice of magnetic material, while competing or interrelated degrees of freedom often obfuscate fundamental material insights. By contrast, magnetic heterostructures and coherent methods for spin control offer a wealth of possibilities for tuning the properties of the THz currents while providing important insight into new interfacial and nonlinear phenomena that are often tied to specific material properties. As such, researchers have largely pivoted to these approaches to leverage magnetic materials for THz generation and other applications.

The development of new THz technologies based on heterostructure architectures and/or nonlinear mechanisms has led to a variety of new insights into underlying current conversion mechanisms, broken symmetries, and fundamental material properties. In heterostructure approaches, laser-driven spin currents are converted into charge currents through so-called inverse processes. Here, interfacial ${\mathscr{P}}$ symmetry breaking becomes an important factor, with potentially strong connections to the structure of the sample and the polarization properties of the laser pulse. As such, inverse processes represent one of the best methods to gain control over THz spin dynamics through symmetry engineering with magnetic materials. Another approach lies in the direct coherent excitation of magnon modes intrinsic to the magnetic material. The ensuing collective magnetization dynamics can drive the emission of THz radiation either directly or in concert with inverse processes, both of which reveal strong connections to the structural symmetry of the material. In the following sections, we will discuss the various magnetically-based THz emission phenomena, exploring them from a mechanistic perspective to gain physical insight into the processes and their link to the underlying material properties (Table 1).

**Table 1:** Overview of magnetic mechanisms and relevant symmetries

Mechanism	Common structure	B field sensitivity	Pump polarization sensitivity	Broken symmetries
Inverse spin-Hall effect (ISHE)	FM/NM	Yes, through FM layer magnetization	No	${\mathscr{P}}$ (interface) ${\mathscr{T}}$ (FM) $\to$ separated ${\mathscr{P}}{\mathscr{T}}$
Inverse Rashba-Edelstein effect (IREE)	FM/NM1/NM2	Yes, through FM layer magnetization	No	${\mathscr{P}}$ (NM1/NM2 interface) ${\mathscr{T}}$ (FM) $\to$ separated ${\mathscr{P}}{\mathscr{T}}$
Inverse spin–orbit torque (ISOT)	FM/NM	Yes, through the FM layer magnetization	Phase change depending on circular polarization state	${\mathscr{P}}$ & ${\mathscr{T}}$ (interface) $\to$ local ${\mathscr{P}}{\mathscr{T}}$
Coherent magnons	AFM	Possible, depending on material	Yes, specifics depend on structure	${\mathscr{T}}$

### Inverse spin-Hall effect

**Fig. 4:**
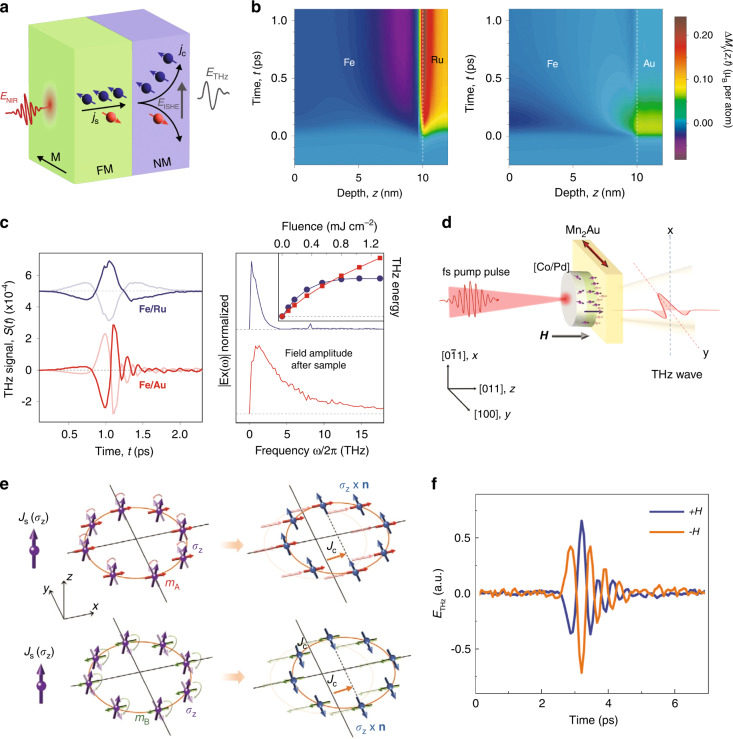
Inverse spin-Hall effect-based THz emission. **a** Schematic of an ISHE-based THz emitter, which converts the ultrafast laser-induced spin current in the FM layer into a charge current in the NM layer. **b** Contrasting interfacial spin accumulation from Ru and Au metallic layers showing the comparatively rapid equilibration in the Au system. **c** THz radiation from Fe/Ru and Fe/Au emitters showing the dramatically larger emission bandwidth of the latter. **d** Schematic of THz emission from [Co/Pd]/Mn_2_Au structure where the spin-polarized current is generated in the Co/Pd layer and the sublattice-mediated reorientation of spins in the AFM Mn_2_Au generates a THz pulse polarized along the direction of magnetization. **e** Illustration of the spin-to-charge current conversion in an AFM, which drives a spin reorientation of out-of-plane polarized spins into antiparallel in-plane direction in the two magnetic sublattices yielding a charge current due to shifting of the Fermi contour. **f** THz transient from the [Co/Pd]/Mn_2_Au emitter showing phase reversal under the change of external magnetic field orientation. Panels (**b**, **c**) reprinted with permission from ref. ^53^ © 2013 Nature Publishing Group. Panels (**d**–**f**) reprinted with permission from ref. ^129^ © 2022 Wiley-VCH GmbH

Intriguingly, when a thin FM metallic film is combined with a non-magnetic (NM) layer in a heterostructure configuration, the THz emission intensity is dramatically enhanced, far beyond what is expected from demagnetization in the magnetic layer alone^53,122^. Notably, in addition to transiently demagnetizing the FM film, the thermal gradient from the laser-induced heating of the FM film can lead to different charge flow for the majority and minority carriers due to their different Seebeck coefficients and a net spin current^123^, ${{\bf{j}}}_{{\rm{s}}}$, which drives the transport of spin polarization into the NM film. The enhancement of THz emission in FM/NM heterostructures is attributed to this spin current through the inverse spin Hall effect (ISHE)^124^, which is schematically represented in Fig. 4a. Here, hot electrons of opposite spin undergo deflections in opposing directions in the NM layer due to the spin-orbit interaction. This yields a charge current, ${{\bf{j}}}_{{\rm{c}}}=\tfrac{{\rho }_{{\rm{sH}}}}{\rho }{{\bf{j}}}_{\rm{s}}\times \hat{{\bf{m}}}$, where $\hat{{\bf{m}}}$ is the magnetization unit vector of the FM layer, and ${\rho }_{{\rm{sH}}}$ and $\rho$ are the spin Hall and longitudinal resistivity of the NM material, respectively. In analogy to photoconductive devices, the picosecond-scale time variation of ${{\bf{j}}}_{{\rm{c}}}$ results in the emission of THz radiation, whose polarity can be switched by reversing the magnetization of the FM layer with an external magnetic field. Accordingly, THz emission via the ISHE requires the breaking of ${\mathscr{T}}$ symmetry by spontaneous magnetic ordering in the FM and ${\mathscr{P}}$ symmetry at the FM/NM interface. As the spin-to-charge conversion process occurs exclusively in the NM layer, spatially separated ${\mathscr{P}}{\mathscr{T}}$ symmetry breaking is responsible for the photocurrent.

A heavy metal is generally utilized for the NM layer, as the efficiency of the ISHE is dependent on strong spin-orbit coupling, scaling as the fourth power of the atomic number^125^. In addition, the spectrum of the THz pulse generated via the ISHE is highly sensitive to the orbital character of the electronic states in the NM material species. In particular, interfacial spin accumulation can be significantly inhibited for states with higher band velocities and carrier lifetimes (e.g., those with *sp* character), as seen in Fig. 4b comparing devices with Ru and Au metallic layers^53^. This leads to more rapid magnetization dynamics and recovery toward equilibrium in the NM layer, resulting in faster charge current dynamics and broader THz emission bandwidths, as seen in Fig. 4c. As such, heterostructures comprised of noble metal films are typically favored for such spintronic THz emitters. Moreover, due to limited spin accumulation, they are found to respond nearly linearly in their THz emission amplitude with respect to the optical pump fluence. Further enhancements in emission amplitude can be achieved by tuning the FM layer to maximize the spin-polarization of the conduction electrons^126^, improving interfacial quality^121^, and optimizing the heterostructure geometry to maximize spin-to-charge current conversion^127^. Notably, the latter has led to the development of tri-layer spintronic devices capable of generating THz pulses with nearly 30 THz of bandwidth and amplitudes rivaling those from optical rectification in ZnTe and GaP crystals.

Finally, we note that the ISHE is not exclusively limited to heterostructures comprised of FM layers. In fact, partially compensated ferrimagnetic (FIM) materials, such as CoGd, have also been shown to support robust THz emission whose phase can be switched by an external magnetic field^128^. Here, the net spin polarization, rather than the net magnetization (which is near zero), is of greater importance. This is because the laser-induced superdiffusive spin current in CoGd is dominated by spin-split Co bands which are close to the Fermi level. More recently, THz emission has also been demonstrated in antiferromagnetic (AFM) heterostructures comprised of Mn_2_Au/[Co/Pd] (Fig. 4d)^129^. In contrast to FM/NM or FIM/NM emitters, here the [Co/Pd] layer supplies an out-of-plane polarized spin current upon photoexcitation. As shown in Fig. 4e, the AFM Mn_2_Au layer drives the spin-to-charge current conversion due to the rotation of the injected spins into the plane of the heterostructure in opposing directions at the two magnetic sublattices by the AFM moment. This leads to a shift of the Fermi contours for the two sublattices, yielding a charge current that generates a THz pulse. As with other spintronic devices based on the ISHE, the THz phase can be reversed by varying the orientation of the external magnetic field (Fig. 4f).

### Inverse Rashba-Edelstein effect

In a metallic heterostructure, ${\mathscr{P}}$ breaking at the interface can also lead to localized states that experience an effective electric field, ${{\bf{E}}}_{{\rm{eff}}}$, normal to the interface in the Rashba-Bychkov model^130^. As such, an electron moving with wavevector ${{\bf{k}}}_{\parallel }$ along the interface experiences an effective magnetic field proportional to $\hslash {{\bf{k}}}_{\parallel }\times {{\bf{E}}}_{{\rm{eff}}}$. Coupling of the electron spin to this field gives rise to an effective interaction Hamiltonian, ${{\mathscr{H}}}_{{\rm{R}}}={\alpha }_{R}\hat{{\boldsymbol{\sigma }}}\cdot \left({{\bf{k}}}_{\parallel }\times \hat{{\bf{n}}}\right)$, where ${\alpha }_{{\rm{R}}}$ is the Rashba coefficient, $\hat{{\boldsymbol{\sigma }}}$ is the Pauli spin matrix, and $\hat{{\bf{n}}}$ is a unit vector normal to the interface.

**Fig. 5:**
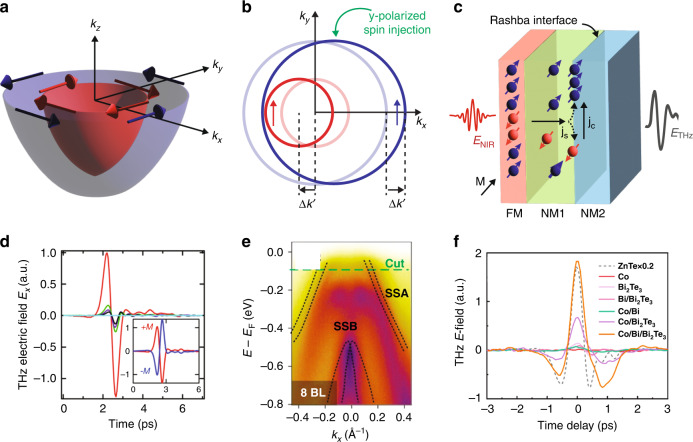
Inverse Rashba-Edelstein effect-based THz emission. **a** The effective field due to the Rashba interaction splits the electronic bands and leads to opposite winding of spin texture in the inner and outer Fermi contour. **b** Spin-polarized injection shifts the Fermi contours in the blue and red bands in (**a**) by $\Delta {k\text{'}}$, leading to a charge current proportional to the injected spin current. **c** Schematic of an IREE-based THz emitter where a spin-polarized current from the FM is converted to a charge current at the NM1/NM2 interface due to the Fermi contour shift depicted in panel (**b**). **d** THz emission from CoFeB/Ag/Bi (red), CoFeB/Bi (green), CoFeB/Ag/Al (purple), CoFeB/Al (black), and MgO/Ag/Bi (cyan) heterostructures with the inset showing the reversal of the THz pulse phase under a 180 degree change in the magnetization direction of the FM layer. **e** Angle-resolved photoemission spectrum of the Rashba-mediated Dirac surface states in Bi/Bi_2_Te_3_ heterostructures. **f** THz emission from various heterostructures highlighting the increased response for Co/Bi/Bi_2_Te_3_ due to the presence of Rashba-mediated splitting of a Dirac surface state at the Bi/Bi_2_Te_3_ interface. Panel (**d**) reprinted with permission from^54^ © 2018 American Physical Society. Panels (**e**) and (**f**) reprinted with permission from ref. ^134^ © 2020 American Chemical Society

For a nearly-free electron gas, the Rashba interaction leads to an offset of $\Delta k=\tfrac{{m}^{* }{\alpha }_{{\rm{R}}}}{{\hslash }^{2}}$ in momentum space between the two opposing spin bands. This spin splitting results in a tangential winding spin texture of the electronic states in momentum space (Fig. 5a). Accordingly, when a spin current is injected towards the interface, for example polarized along the $+y$ direction, the population on one side of the Fermi contour increases while the other side decreases, as depicted in Fig. 5b, effectively shifting the contours in momentum space by some $\Delta {k\text{'}}$. This nonequilibrium state drives a charge current density that is proportional to the spin current, a phenomenon known as the inverse Rashba-Edelstein effect (IREE). As such, THz emission results from separated ${\mathscr{T}}$ symmetry breaking in the FM layer and ${\mathscr{P}}$ symmetry breaking at the NM1/NM2 interface. However, unlike the ISHE, the spin-to-charge conversion is entirely restricted to the interface.

Following early studies at microwave frequencies^131,132^, femtosecond optical pumping has recently yielded charge currents capable of driving THz emission in FM/Bi/Ag heterostructures^54,55^, schematically represented in Fig. 5c. This is due to the intrinsically large spin splitting in Ag/Bi interfaces^133^. The emission amplitude of the tri-layer structures were found to be nearly 2–3 orders of magnitude larger than FM/Bi, FM/Ag, and other control structures lacking the Rashba interface (Fig. 5d). Furthermore, the emitted THz amplitude was found to be highly sensitive to the thickness of the Bi layer^55^. This is likely due to effects such as the decay of the injected spin current and multiple reflection effects when it makes up the second and third layers, respectively. THz emission from the IREE has also been demonstrated with topological insulators using FM/Bi/Bi_2_Te_3_ heterostructures^134^. As shown via angle-resolved photoemission spectroscopy (ARPES; Fig. 5e), Rashba-mediated splitting of a Dirac surface state manifests as spin-split bands located outside the Dirac cone with a strong spin polarization. These originate from a hybridization of Rashba surface state from a bismuth film with a topological surface state in Bi_2_Te_3_. The THz emission amplitude, shown in Fig. 5f for different bilayer and trilayer structures, is largest for Co/Bi/Bi_2_Te_3_ owing to the Rashba-split Dirac surface state. The emission amplitude increases with Bi thickness, peaking at 7 bilayers when the spin-split bands become a real surface state (Fig. 5e), with the THz intensity nearing that emitted from a 1-mm thick ZnTe crystal.

### Inverse spin–orbit torque

**Fig. 6:**
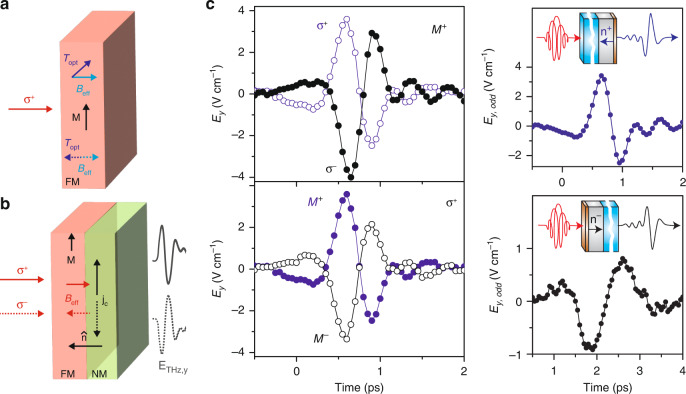
THz emission via inverse spin–orbit torque effect. **a** Schematic of spin-orbit torques that can result from effective magnetic fields driven by an ultrafast pump pulse. **b** Pump helicity dependence of THz emission via ISOTE, where different circular polarization states give rise to different orientations of optically-induced effective magnetic fields and THz pulse phase reversal. **c** THz transient from a Co/Pt heterostructure showing phase reversal under interchange of pump helicity, sample, magnetization, and layer order. Panel (**c**) adapted with permission from ref. ^137^ © 2016 Nature Publishing Group

In the processes described thus far, the polarization state of the driving optical pulse is largely immaterial, while the polarization of the emitted THz pulse is linear with its orientation and phase dictated exclusively by the magnetization vector in the FM layer. However, deterministic all-optical control of the THz pulse phase can also be achieved through the inverse spin-orbit torque effect (ISOTE). When a circularly polarized optical pulse impinges on an FM sample, the spin-orbit interaction can yield a pump-induced torque on the spin system (Fig. 6a). The orientation of this torque depends on the relative orientation of the magnetization of the material and the optically-induced effective magnetic field, which can arise from inverse Faraday and optical spin-transfer effects^135,136^. The ensuing optically-induced spin reorientation in an FM can be converted to a charge current in an FM/NM heterostructure, where ${\mathscr{P}}$ symmetry breaking at the interface drives an interfacial spin photocurrent, ${\bf{j}}\propto \hat{{\bf{n}}}\times \left[\hat{{\bf{m}}}\times {{\bf{B}}}_{{\rm{eff}}}\right]$^136^. Here, $\hat{{\bf{n}}}$ is a unit vector along the direction of broken inversion symmetry (pointing from the FM to the NM layer), $\hat{{\bf{m}}}$ is the magnetization unit vector of the FM layer, and ${{\bf{B}}}_{{\rm{eff}}}$ is an effective magnetic field whose direction is parallel or antiparallel to the laser pulse propagation direction for right and left circularly polarized light, respectively. In contrast to the ISHE and IREE, while ${\mathscr{P}}$ and ${\mathscr{T}}$ symmetries are broken within the FM and at the interface, respectively, the local ${\mathscr{P}}{\mathscr{T}}$ breaking induced by ${{\bf{B}}}_{{\rm{eff}}}$ at the interface is critical to charge current generation in the ISOTE. The rapidly changing photocurrent leads to THz emission, as depicted in Fig. 6b, with a phase that can be reversed by changing the optical pulse helicity, FM layer magnetization direction, and/or the spatial ordering of the FM and NM (which changes the sign of $\hat{{\bf{n}}}$).

THz emission through the ISOTE was first realized in Co/Pt heterostructures, similar to those used in spintronic emitters based on the ISHE^137^. A key difference is that the THz emission based on the ISOTE is linearly polarized along the FM layer’s magnetization vector, while the ISHE contribution is orthogonally oriented. This allows the contributions due to the ISHE and ISOTE to be separated, with the latter being approximately 7-fold weaker. As shown in Fig. 6c, the THz field due to the ISOTE from Co/Pt heterostructures possesses the characteristic phase reversal under a change of pump helicity, sample magnetization, and layer order. Similar THz emission has been observed in FeRh/Pt heterostructures^138^ as well as the FM/NM/NM heterostructures used in investigations of the IREE, but were conspicuously absent in FM/NM control devices^54^. Notably, the relative strength of the helicity-dependent THz emission in the FM/NM/NM structures was nearly five-fold weaker than in Co/Pt structures. Ultimately, ISOTE-based THz emission appears to be a far less ubiquitous phenomenon, leading to some debate regarding the strength of this mechanism. Recent investigations into the impact of interfacial properties on the strength of ISOTE-based THz emission may explain these conflicting findings^121^. In particular, increased surface roughness at the interface enhances the relative efficiency of ISOTE-based THz emission. This is attributed to a thicker effective FM/NM interface (i.e., greater contact area between the FM and NM), which yields a larger charge current. In fact, heterostructures with intermixing layers (e.g., Co/Co_x_Pt_1-x_/Pt) show significantly less helicity dependence of the THz emission, emphasizing the importance of contact area between the pure FM and NM layers.

### THz emission from rotational symmetry in AFMs

**Fig. 7:**
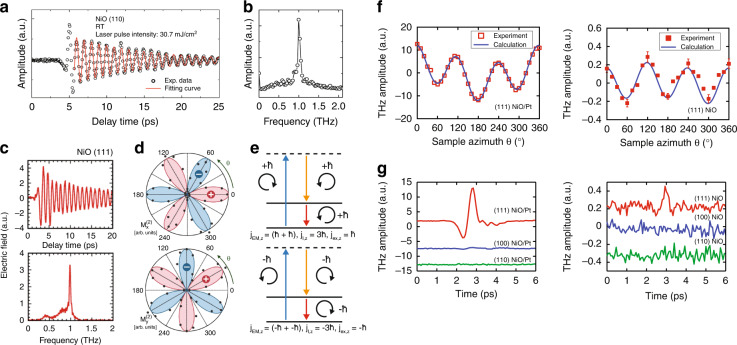
THz emission from rotationally symmetric magnetic systems. **a** THz emission from (110) NiO driven by a near-infrared ultrafast pulse via ISRS and (**b**) the corresponding Fourier transform. **c** THz emission from (111) NiO driven by a near-infrared ultrafast pulse via ISRS (upper panel) and corresponding Fourier transform (lower panel). **d** Six-fold rotational symmetry of $x$- (upper panel) and $y$-polarized (lower panel) THz field components from (111) NiO under sample rotation, where red lobes correspond to a positive signed transient and blue lobes correspond to a negative signed transient. **e** ISRS selection rules for colinear scattering where the upward blue arrow indicated photon annihilation, the downward orange arrow indicates photon creation, and the downward red arrow indicates magnon excitation. **f** Six-fold symmetric THz emission from (111) NiO/Pt heterostructure (left panel) and (111) NiO (right panel) under sample rotation showing dramatic enhancement in the heterostructure. **g** THz transients from NiO/Pt heterostructures with various NiO crystal faces (left panel) and corresponding bare NiO response. Panels (**a**) and (**b**) reprinted with permission from^143^ © 2010 American Institute of Physics. Panels (**c**) and (**d**) reprinted from^144^ © 2011 American Physical Society, under the terms of CC-BY 3.0. Panels (**f**) and (**g**) reprinted with permission from^145^ © 2020 the Author(s), under exclusive licence to Springer Nature

Thus far, we have primarily focused on THz emission through demagnetization and inverse effects. However, the excitation of spin resonances can also lead to the emission of THz pulses. An optical pulse with sufficient bandwidth can contain many photon pairs whose energy difference is equivalent to that of a vibrational or magnetic mode in the crystal. If the mode is Raman active, such a pulse can be used as a coherent drive through a mechanism known as impulsive stimulated Raman scattering (ISRS)^139^. If the mode is also dipole active, the coherent oscillation can also drive the emission of electromagnetic radiation through dipole processes. This was observed in a number of compounds^140–142^, notably (110) NiO, where linearly polarized near-infrared femtosecond pulses with photon energy below the bandgap yielded THz radiation with a highly oscillatory signature (Fig. 7a), in stark contrast to the nearly single-cycle emission from spintronic systems discussed above^143^. The Fourier transform (Fig. 7b) reveals that the oscillatory component has a frequency that coincides with the AFM magnon resonance of approximately 1 THz, with a temperature dependence in close agreement with theory. The simplicity of the THz emission process from coherent magnons belies its potential link to the underlying symmetry of the AFM crystal. As seen in Fig. 7c for (111) NiO, linearly polarized photoexcitation yields similar emission of THz radiation with a $\sim$1 THz frequency^143^. However, the $x$- and $y$-polarized components each have a characteristic six-fold intensity amplitude modulation under sample rotation (Fig. 7d)^144^. Even more striking is the absence of any THz emission when the ultrafast pump is circularly polarized. As we will discuss next, this is linked to the three-fold rotational symmetry of (111) NiO.

In a coherent optical process such as ISRS, a time-varying magnetization can be driven as a nonlinear difference frequency generation process,

6 $${M}_{i}^{\left(2\right)}\left(\Omega \right)={\chi }_{{ijk}}^{\left(2\right),{\rm{MEE}}}\left(\Omega {\rm{;}}{\omega }_{1},-{\omega }_{2}\right){E}_{j}\left({\omega }_{1}\right){E}_{k}^{* }\left({\omega }_{2}\right)$$

where ${\chi }_{{ijk}}^{\left(2\right),{\rm{MEE}}}$ is the nonlinear susceptibility, ${E}_{j,k}$ are the electric field components of the ultrafast pump pulse, $\Omega$ is the frequency of the excitation, and ${\omega }_{\mathrm{1,2}}$ are the frequencies of the photon pair constituents in the incident ultrafast pump. We can understand the role of rotational symmetry from the perspective of angular momentum (${\bf{J}}$) conservation, which in general has contributions from the electromagnetic field, ${{\bf{J}}}_{{\rm{EM}}}$, the excitation, ${{\bf{J}}}_{{\rm{ex}}}$, and the crystal lattice, ${{\bf{J}}}_{{\rm{l}}}$. These must be in balance, such that ${{\bf{J}}}_{{\rm{EM}}}+{{\bf{J}}}_{{\rm{ex}}}+{{\bf{J}}}_{{\rm{l}}}=0$. This has important consequences for a driving ultrafast pulse propagating along the rotational symmetry axis (labeled $z$) of a crystal. In the case of ${C}_{3}$ symmetry, ${J}_{{\rm{EM}},z}+{J}_{{\rm{ex}},z}$ need only be conserved to within $3\hslash$, by analogy with traditional Umklapp scattering processes. This leads to selection rules for the ISRS (Fig. 7e), where the annihilated and created (or, alternatively, the incident and scattered) photons are of opposite helicity with ${J}_{{\rm{EM}},z}=2\hslash$. Due to the three-fold symmetry, we can freely let ${J}_{{\rm{l}},z}=3\hslash$, inducing a magnon excitation with ${J}_{{\rm{ex}},z}=\hslash$, emitting a left circular photon, and satisfying conservation constraints. This can be achieved with a linearly polarized pump pulse and an equivalent process involving ${J}_{{\rm{l}},z}=-3\hslash$ is also possible. When these two are balanced, the result is linearly polarized THz emission at the magnon excitation frequency.

The dynamic magnetization manipulation afforded by difference frequency generation can also be exploited to produce broadband THz emission by combining it with ISHE-based spin-to-charge current conversion. As shown in Fig. 7g, NiO/Pt heterostructures photoexcited with linearly polarized ultrafast pulses were shown to generate near single-cycle THz emission via the ISHE. Curiously, a characteristic six-fold pattern with respect to sample rotation, similar to the case of narrowband emission from bulk (111) NiO samples, was also observed (Fig. 7f)^145^. The similar symmetry but strikingly different bandwidth of the THz emission can be attributed to a second difference-frequency-based radiative mechanism. In addition to the magnetic dipole THz emission discussed earlier, the dynamic change in the magnetization can also give rise to a spin current of the form

7 $${j}_{{\rm{s}},i}\propto {M}_{i}^{\left(2\right)}\left(t\right)=\int \,d{\omega }^{{\prime} }{\rm{exp }}\left(i{\omega }^{{\prime} }t\right)\int d\omega {\chi }_{{ijj}}^{\left(2\right),{\rm{MEE}}}\left({\omega }^{{\prime} }{\rm{;}}{\omega }^{{\prime} }-\omega ,\omega \right){E}_{j}^{* }\left({\omega }^{{\prime} }-\omega \right){E}_{j}\left(\omega \right)$$

As in the FM/NM heterostructures described above, this spin current can be converted to a charge current via the ISHE in the Pt layer, leading to the emission of the THz pulse. This also explains the six-fold symmetry of the THz amplitude, which originates from the rotational symmetry of the spin current stemming from ${\chi }^{\left(2\right),{\rm{MEE}}}$. It should be noted that this type of spin current generation follows purely from the three-fold rotational symmetry of (111) NiO. Therefore, as shown in Fig. 7g, similar heterostructures comprised of either (110) or (100) NiO did not show significant THz emission since in the latter two, the normal vector is no longer the axis of rotational symmetry. The ability to confer polarization sensitivity on ISHE-based THz emission represents a significant new advancement in our understanding of magnetically driven processes and our ability to harness AFM materials for more advanced spintronic applications, with greater control of the THz emission properties. This is especially true as difference-frequency-based spin current generation in crystals with three-fold rotational symmetry is likely to be a generic phenomenon in AFM insulators and can be applied to a variety of existing spintronic systems.

## Designer symmetries in low-dimensional systems

Micro- and nano-scale structuring introduces a variety of new physical properties beyond those available in the bulk, including new spatial symmetries that enhance or even fundamentally induce THz emission. While a remarkable breadth of physics and chemistry occurs at relatively simple interfaces, more exotic physical properties and dynamics can emerge as the dimensionality of a material is reduced, particularly when additional translational and rotational symmetries are introduced via stacking, patterning, and twisting. This allows for the manipulation of energy-momentum dispersion in artificial photonic^146^, electronic^147,148^, and acoustic/optomechanical^149^ crystals, as well as spatially varying anisotropic effective medium properties of metamaterials^150^. In metasurfaces, for instance, plasmonic and dielectric meta-atoms (nano/micro-resonators) can be designed with resonances ranging from visible to microwave frequencies. Tailoring such structures within the THz range has led to new levels of control over THz radiation, including broadband polarization conversion^151,152^, narrowband frequency filtering^153^, and active filtering/modulation^154–156^, as well as a variety of functionalities associated with the control of spatially-dependent amplitude/phase profiles^157^. In these artificial materials, the resonator geometries and corresponding multipolar responses yield local radiation patterns that interfere in the far field for desired global responses, depending on the overall spatial arrangement of the meta-atoms.

More generally, plasmonic systems—including metals, semimetals, and doped semiconductors—offer extended spatiotemporal control over nanoscale dynamics across photonic, electronic, phononic, and thermal degrees of freedom. Controlling the momentum distributions of hot carriers is a particularly important area of investigation^158^, with THz emission spectroscopy offering new opportunities for studying the resulting ultrafast charge dynamics^159^. Conversely, controlled hot carrier momentum flows in hybrid plasmonic systems can yield tailored THz radiation fields, beyond the resonant field enhancement effects that have been exploited in photoconductive antennas^160^. Plasmonics is therefore a significant frontier area for THz science, and vice versa.

We thus begin this section by considering evolving insights from ultrafast THz emission studies of flat, randomly structured, and nano-patterned metal surfaces, culminating in two emerging designer systems: plasmonic nanocathode arrays with optically controlled nonlinear photoelectron currents emitted into free space (or a nearby material), and plasmonic metasurfaces with rectified currents within the metal structures. Extending out in dimensionality, we then review recent insights on 1D carbon nanotubes and semiconductor nanowires, 2D materials/heterostructures, and hybrid nanostructure–2D systems studied via THz emission spectroscopy.

### Nanostructured metal surfaces

Studies of THz emission from non-magnetic metal surfaces were originally performed with the goal of generating intense THz radiation using amplified optical pulses^161,162^. Although largely eclipsed by more efficient and/or broadband tilted-pulse-front LiNbO_3_^163^, air plasma^164^, and spintronic^53,59^ sources^165^, nominally smooth gold and silver films nevertheless display several intriguing behaviors that have stimulated a significant body of work. Perhaps the most surprising observation has been the deviation from the second-order fluence dependence, ${\Phi }_{{\rm{THz}}}\propto {\Phi }_{0}^{2}$, which is expected for many THz emission mechanisms. In the initial studies of Kadlec et al.^161,162^, for instance, a higher-order fluence dependence (${\Phi }_{{\rm{THz}}}\propto {\Phi }_{0}^{n}$ with $n \,>\, 3$) gave way to apparent second-order dependence with increasing fluence. Yet it is unexpected that a lower-order process should overtake a higher-order process with increasing pulse fluence.

As with the optical rectification that occurs due to bound charge oscillations in the anharmonic potential at the surface of an insulator, low-frequency (including THz-range) nonlinear polarization fields are generated at flat surfaces of centrosymmetric metals due to ${\mathscr{P}}$ symmetry breaking at the interface. Incident p-polarized femtosecond laser fields drive out-of-plane oscillations of the free conduction electrons as well as bound electrons (e.g., filled $d$-band for noble metals), with second harmonic components around $2\omega$ and rectified components in the low-frequency limit. The free electron contribution has been modeled hydrodynamically for second harmonic generation^166,167^, yielding

8 $${{\mathbf{j}}}_{{\mathrm{S}}}\left(2\omega \right)=-2i\omega \left(\beta {\bf{E}}\left(\nabla {{\cdot}}{\mathbf{E}}\right)+\gamma \nabla \left({\mathbf{E}}{{\cdot}}{\mathbf{E}}\right)\right)$$

in which $\beta =\tfrac{e}{8\pi {m}^{* }{\omega }^{2}}$ and $\gamma =\tfrac{{e}^{3}{n}_{e}}{8{m}^{* }{\omega }^{4}}$ are derived, although these coefficients are often treated phenomenologically to accommodate unknown surface quality. The first term in Eq. 8 is known as the surface contribution, due to the field discontinuity at the surface boundary layer, while the second term, due to the field intensity decay within the penetration depth of the medium, is referred to as the bulk contribution. In both cases the dominant current component is normal to the interface. A similar expression was later derived for sum and difference frequency generation at metal surfaces^168^, as relevant to THz emission. The roles of atomic orbital symmetries for $d$-band transition metals versus ${sp}$-band noble metals have also been considered^169^, with ${\chi }_{{zzz}}^{\left(2\right),{\rm{S}}}$, ${\chi }_{{zxx}}^{\left(2\right),{\rm{S}}}$, and ${\chi }_{{xzx}}^{\left(2\right),{\rm{S}}}$ allowed for noble metals ($z$ normal to the surface, $x$ along the reflection plane) but with the latter in-plane contribution found to be small in initial THz emission experiments^162^. Subsequently, in-plane currents have been observed in higher-fluence studies (>10 mJ cm^-2^), attributed to the combined action of the in-plane field component accelerating the charged density induced by the surface-normal field component^170^, corroborated by the incident angle dependence for p-polarized optical pulses^171^. However, the observation of exponential fluence dependence in these studies is also suggestive of thermal mechanisms. Subsequent theoretical work has proposed additional metal surface THz generation mechanisms, ranging from asymmetric heating to ponderomotive and photon drag effects^172–174^, with an emphasis on high-intensity s-polarized radiation.

**Fig. 8:**
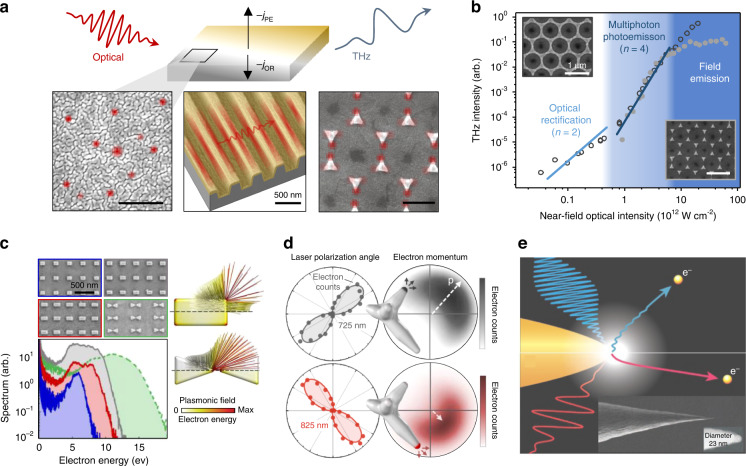
Terahertz emission from nanostructured metal surfaces and plasmonic nanocathodes. **a** Localized and traveling plasmonic modes and illustrations of corresponding optical hot spots on (left to right) percolated ultrathin ($\sim$10 nm) films, nanogratings, and nanopatterned arrays. **b** Dependence of THz intensity on plasmon-enhanced near-field optical intensity for two different Ag nanostructure arrays, estimating a 7.5-fold near-field enhancement for the nanohole array (open circles) and 20-fold enhancement for the nanotriangle array (solid circles). Vertical offsets adjusted for clarity. Plasmonic **c** nanostructure, **d** nanoparticle, and **e** etched nanotip photocathodes with controlled photocurrent directionality and multiphoton to strong-field emission (along with post-emission ponderomotive dynamics). Panel **a** left inset (Ag thin film) and right inset (Ag nanotriangle array) adapted with permission from^178^ © 2011 American Chemical Society. Panel **a** middle inset (Au nanograting) adapted with permission from^176^ © 2011 Springer-Verlag. Panel **b** adapted with permission from^159^ © 2014 American Physical Society. Panel **c** adapted with permission from^185^ **©** 2013 American Chemical Society. Panel **d** adapted from^189^ © 2020 under the terms of CC-BY 4.0. Panel **e** adapted with permission from ref. ^191^ © 2012 Nature Publishing Group

Following the initial work on nominally flat metal films, subsequent investigations found enhanced THz emission due to nanoscale structuring, including with gratings^175–177^, randomly-distributed nanostructuring^178–182^, and precision lithographically-defined nanostructure arrays^159,178^ (Fig. 8a). These systems introduce new geometry-dependent light-matter interactions, which manifest in the strong spatially varying plasmonic field enhancements. Whether traveling surface plasmon-polariton modes excited on gratings, localized nanostructure resonance modes, or contributions from both on percolated ultrathin metal films, the deeply sub-diffractive concentration of optical electric fields within plasmonic systems are enabled by the partitioning of energy between the electric field and free carrier momentum (rather than magnetic field) harmonic oscillator quadratures^183^. Field intensity enhancements in plasmonic “hot spot” regions can exceed 10^3^, while nanostructure geometry determines the spatial distribution of such hot spots in response to incident optical fields with different polarization and frequency.

Beyond enhanced THz emission, the greater level of control over field enhancements in these nanostructured systems has also elucidated the mechanisms underlying higher-order fluence dependence, with multiphoton photoemission and post-emission ponderomotive acceleration suggested for studies of gold gratings^175^ as well as controlled percolated silver films and nanostructure arrays^178^. However, the relative roles of optical rectification and multiphoton photoemission were only clarified by Polyushkin et al. ^159^ in combined THz emission and photoelectron emission studies of nanostructure arrays. A low-order THz emission fluence dependence (consistent with optical rectification) was demonstrated at lower incident fluences, transitioning to higher-order ($n\approx$ 4) behavior at intermediate fluences, followed by a leveling off to apparent low-order behavior again at the highest fluences (Fig. 8b). This high-fluence transition was also evident in the photoemission data and marked a well-known transition into the optical field emission regime, in which the perturbative multiphoton expansion is no longer valid and strong-field tunneling emission takes over. Such transitions can be characterized by the dimensionless Keldysh parameter^184^, $\gamma =\tfrac{\omega }{{\omega }_{t}}$, where ${\omega }_{t}=\tfrac{{eE}}{\sqrt{2m\phi }}$ is the frequency associated with the electron tunneling time through the triangular surface barrier, influenced by the electric field ($E$) and work function ($\phi$). Multiphoton photoemission is dominant in the low-field, high-frequency regime ($\gamma \gg 1$) and field emission is dominant in the high-field, low-frequency regime ($\gamma \ll 1$). In the photoemission data of Polyushkin et al.^159^, a transition is indeed observed for input intensities around 30 GW cm^−2^ corresponding to a transitional $\gamma \approx$ 1.5, estimating a 20$\times$ plasmonic field enhancement (peak near-field intensity ~10^13^ W⋅cm^-2^).

Characterization of the THz emission mechanisms in many of these systems has been complicated by surface roughness and contamination (particularly for silver^177^), but the processes leading to THz emission from metal surfaces can be approximately summarized as (Fig. 8b): (i) optical rectification for low excitation pulse fluences (laser oscillator range), (ii) multiphoton photoemission for intermediate fluences (from oscillators or high-repetition amplifiers), and (iii) optical field emission for high fluences (low-repetition amplifiers), along with potential thermal or thermally-assisted effects. The transitional intensities are highly dependent on material work function, excitation frequency, and roughness- or structure-induced local field enhancements. THz signals in the optical rectification regime may be observed in the low-fluence limit of amplified pulse measurements^178^, while plasmonic field enhancements allow for observations of field emission with oscillators^185^, and multiphoton photoemission has even been observed recently with continuous-wave light^186^.

Although photoemission spectroscopies serve as powerful tools for studying emitted electron distributions and dynamics, THz emission spectroscopy provides new capabilities for studying the ultrafast dynamics of charges moving within materials (internal photocurrents) and in free space (external photocurrents), with the added benefit of operating at ambient pressures^178^ rather than at high or ultrahigh vacuum. Furthermore, THz radiation directly reflects the near-field multiphoton/field emission photocurrent dynamics, whereas these dynamics are only indirectly inferred from the final kinetic energy spectra in photoemission spectroscopies. Thus, THz emission can be expected to offer valuable insights in emerging studies of plasmonic nanocathodes^185,187–191^ (Fig. 8c–e), which provide a new degree of control over nanoscale, ultrafast photocurrent momentum distributions in free-space and nearby materials. Square gratings exhibit in-plane centrosymmetry (Fig. 8a), as do randomly structured thin films on average, yielding only out-of-plane dipoles that benefit from plasmon-enhanced fields yet reproduce the basic symmetry of a flat metal interface. In other words, despite the local ${\mathscr{P}}$ symmetry breaking at the interfaces, the overall in-plane centrosymmetry of these sub-wavelength structures preserves in-plane inversion (or equivalently, ${C}_{2}$) symmetry. By contrast, nanostructures can be designed with effectively arbitrary 2D geometries, for polarization- and/or frequency-sensitive plasmonic hot spot excitations, with oriented fields and net in-plane rectification (Fig. 8d). This mapping from optical parameter space onto nanoscale hot spot spatial distributions can be utilized to control the local and global symmetries of the plasmon-enhanced nonlinear optical interactions. Corresponding spatial symmetries in these hot spot regions influence the vector momentum distributions of photoexcited carriers^187,189,190^, leading to new capabilities for optically generating and actively controlling directional transient currents down to the nanoscale. Emerging nanoscopic tip-based THz emission techniques^192–194^ illustrate further opportunities for viewing these THz dynamics at the nanoscale.

### Plasmonic metasurface terahertz emitters

**Fig. 9:**
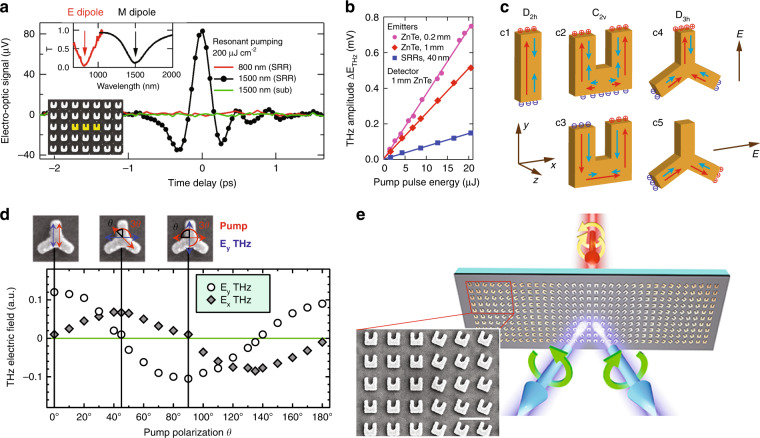
THz emission from nanoplasmonic metasurfaces. **a** THz pulse generated from an array of nanoplasmonic SRRs (bottom inset) excited at different resonance modes (top inset). **b** The field amplitude of the generated THz pulses versus pump power measured with different THz emitters, revealing comparable THz generation efficiency and the second-order nonlinearity. **c** Illustration of a few nanoplasmonic resonators with different symmetries, along with the polarization vectors at excitation (red arrows) and THz (cyan arrows) frequencies. **d** The rotation of THz linear polarization angle as a function of the excitation polarization angle. **e** Spatial separation of left- and right-handed circularly polarized THz radiation by implementing a linear PB phase gradient in the horizontal direction. Panels (**a**) and (**b**) adapted with permission from ref. ^200^ © 2014 Nature Publishing Group. Panel (**d**) adapted from ref. ^207^ © 2021 the Author(s) under the terms of CC-BY 4.0. Panel (**e**) adapted with permission from ref. ^208^ © 2021 American Chemical Society

One motivation of the original proposal of split-ring resonators (SRRs)—the key component for the demonstration of negative-index metamaterials^195,196^— was to facilitate nonlinear responses arising from materials integrated at the critical locations where the local fields were resonantly enhanced by orders of magnitude^197^. Experimental efforts, however, started by observing orders of magnitude enhancement in second harmonic generation arising from the constituent materials comprising the nanostructured SRRs as the source of nonlinearity^198,199^. As optical rectification (difference frequency generation) occurs simultaneously with second harmonic generation (sum frequency generation), emission of much lower THz frequencies through optical rectification becomes possible when nanoplasmonic resonators are excited by femtosecond lasers. Single-cycle broadband THz pulses up to 4 THz were experimentally observed from gold SRRs with few tens-of-nanometers thickness pumped by femtosecond laser pulses at telecommunications wavelengths (Fig. 9a), and the emitted THz electric field exhibited a linear dependence on the pump power (Fig. 9b), thereby corroborating the second-order nonlinearity^200^ (optical rectification). The field intensity of the generated THz radiation from such a thin layer of gold SRRs (40 nm thick) on an indium tin oxide (ITO) coated glass substrate approached that from 5000-fold thicker 200 μm ZnTe crystals (Fig. 9b), revealing a giant effective sheet nonlinear susceptibility. Note that here the optical rectification is essentially a difference-frequency generation process, thus the THz radiation bandwidth is only limited by the pulse duration of the femtosecond laser, providing an opportunity to further increase the THz radiation bandwidth.

One may also conceive further improvement of THz generation efficiency by stacking multi-layer metasurfaces, in which the total thickness could be still sufficiently thin to avoid phase-matching issue. However, efficiently coupling the incident light into a multi-layer metasurface remains a great challenge, as the resonant reflection caused by the large impedance mismatch is dominant at the top layer of the metasurface. In this context, an ultrathin metamaterial absorber structure^201,202^ could provide a viable route, where the incident femtosecond laser pulses are trapped within the metamaterial cavity to continuously excite THz radiation until fully absorbed.

Similar to second-harmonic generation, the THz radiation intensity depends strongly on the symmetry and resonance mode of the plasmonic resonators, and thereby the incident light polarization. We illustrate in Fig. 9c a few example nanoplasmonic unit cells with different symmetries to graphically reveal the THz generation mechanism, with the incident light propagating along the $z$ direction. As the cut-wire resonator (Fig. 9c1) exhibits a ${D}_{2h}$ symmetry with an inversion center, no optical rectification and THz generation is expected (due to cancellation as shown in Fig. 9c1 and discussed below), unless by other mechanisms such as near-field ponderomotive acceleration of surface-plasmon-assisted out-of-plane photoelectrons through multiphoton absorption^175,203^. For the noncentrosymmetric U-shaped SRR with ${C}_{2v}$ symmetry in Figs. 9c2 and 9c3, the relevant nonvanishing tensor elements are^56^ ${{\rm{\chi }}}_{{yxx}}^{(2)}$, ${{\rm{\chi }}}_{{yyy}}^{(2)}$, ${{\rm{\chi }}}_{{xyx}}^{(2)}$, and ${{\rm{\chi }}}_{{xxy}}^{(2)}$. Experimental results with both second harmonic^198,199^ and THz generation^200,204^ revealed that the effective ${{\rm{\chi }}}_{{yxx}}^{(2)}$ (configuration shown in Fig. 9c3) dominates and is enhanced by orders of magnitude.

This behavior can be understood by a hydrodynamic model for the electronic response in nanoplasmonic SRRs, which was introduced first for interpreting second harmonic generation^205^ and then the generation of THz radiation via optical rectification^200^. In this model, the second-order nonlinear THz polarization vector ${{\bf{P}}}_{{\rm{THz}}}\left(\Omega ={\omega }_{2}-{\omega }_{1}\right)\propto a{{\bf{P}}}_{1}^{* }\left({\omega }_{1}\right)\nabla \cdot {{\bf{P}}}_{2}\left({\omega }_{2}\right)+b\left({{\bf{P}}}_{1}^{* }\left({\omega }_{1}\right)\cdot \nabla \right){{\bf{P}}}_{2}({\omega }_{2})$, where ${{\bf{P}}}_{\mathrm{1,2}}$ is the polarization vector of free electrons at excitation frequency ${\omega }_{\mathrm{1,2}}$ within the bandwidth of the pump pulse. In the case of the cut-wire resonator shown in Fig. 9c1, ${{\bf{P}}}_{{\rm{THz}}}$ (cyan arrows) cancels exactly as the gradient (divergence) of ${P}_{\mathrm{1,2}}$ (${{\bf{P}}}_{\mathrm{1,2}}$; red arrow) has an opposite sign at the top and bottom halves of the cut wire. It can be similarly analyzed in the case of electric-dipole resonance in the SRR under $y$-polarized excitation (Fig. 9c2), where ${{\bf{P}}}_{{\rm{THz}}}$ is largely (exactly) canceled in the $y$ ($x$) direction, resulting in negligible $y$-polarized and zero $x$-polarized THz radiation (i.e., negligible ${\chi }_{{yyy}}^{\left(2\right)}$ and vanishing ${\chi }_{{xyy}}^{\left(2\right)}$). By contrast, as shown in Fig. 9c3, $x$-polarized incident light excites a magnetic LC dipole resonance with circulating current oscillation ${{\bf{j}}}_{\mathrm{1,2}}=-i\omega {{\bf{P}}}_{\mathrm{1,2}}$ with oppositely signed gradient (divergence) of ${P}_{\mathrm{1,2}}$ (${{\bf{P}}}_{\mathrm{1,2}}$) at one SRR arm as compared to the other, leading to the same direction of ${P}_{{\rm{THz}},y}$ which oscillates in-phase and constructively emits *y*-polarized THz radiation to the far-field. This results in a resonantly enhanced effective ${\chi }_{{yxx}}^{\left(2\right)}$. Note there is also excitation of ${{\bf{P}}}_{\mathrm{1,2}}$ at the SRR base from left to right, but its gradient has an opposite sign at the left and right sides of the SRR base, leading to the exact cancellation of ${P}_{{\rm{THz}},{\rm{x}}}$. The other two tensor elements (${\chi }_{{xyx}}^{\left(2\right)}$ and ${\chi }_{{xxy}}^{\left(2\right)}$) involve interactions between the $x$- and $y$-polarized incident light. One may take the ${{\bf{P}}}_{\mathrm{1,2}}$ distribution from Fig. 9c2 and the $\nabla \cdot {{\bf{P}}}_{\mathrm{1,2}}$ distribution from Fig. 9c3. Following a similar analysis, it is easy to verify that both effective ${\chi }_{{xyx}}^{\left(2\right)}$ and ${\chi }_{{xxy}}^{\left(2\right)}$ are nonvanishing (but are expected to be small).

In the case of the three-petal resonant nanostructure shown in Figs. 9c4 and 9c5, the ${D}_{3h}$ point group contains a three-fold rotational symmetry and the relevant nonvanishing tensor elements include ${\chi }_{{yyy}}^{\left(2\right)}=-{\chi }_{{yxx}}^{\left(2\right)}=-{\chi }_{{xxy}}^{\left(2\right)}={-\chi }_{{xyx}}^{\left(2\right)}$. Arrays of such three-petal nanoantennas have also been shown to enhance second harmonic generation^206^ and THz radiation^207^. For simplicity, we highlight the first two tensor elements with resonantly excited ${{\bf{P}}}_{\mathrm{1,2}}$ and ${{\bf{P}}}_{{\rm{THz}}}$ in Figs. 9c4 and 9c5. Under *y*-polarized excitation in Fig. 9c4, the *y*-component of ${{\bf{P}}}_{{\rm{THz}}}$ at the top petal dominates, and it is partially canceled by the *y*-component of ${{\bf{P}}}_{{\rm{THz}}}$ at the bottom two petals, resulting in a net ${P}_{{\rm{THz}},{\rm{y}}}$. The *x*-component of ${{\bf{P}}}_{{\rm{THz}}}$ at the bottom two petals, on the other hand, completely cancels each other. Similarly, under *x*-polarized excitation in Fig. 9c5, the *y*-component of ${{\bf{P}}}_{{\rm{THz}}}$ at the bottom two petals adds up, leading to a net ${P}_{{\rm{THz}},{\rm{y}}}$, while the *x*-component completely cancels each other. Thus, in both configurations excited by linearly polarized incident light, the emitted THz radiation is polarized in the $y$ direction but with opposite polarity. This change of THz polarity is associated with the three-fold rotation symmetry of the resonator. Rotating the incident light polarization by $\theta$ leads to $-3\theta$ rotation of the THz polarization with respect to the incident light polarization^207^ (Fig. 9d). Equivalently, rotating the resonator structure by $\theta$ results in the rotation of the THz polarization by $3\theta$. In the case of SRR excited by circularly polarized light, the rotation angle of the THz linear polarization is $\theta$ and it does not depend on the handedness of the excitation light^208^. This offers an opportunity to conveniently control the THz linear polarization direction^207^, and can be further exploited to generate THz vector beams^208^ by appropriately arranging the spatial profile of the resonator orientation, thereby the local THz field polarization direction.

Such a rotation of THz linear polarization by changing the resonator orientation is accompanied by a geometric Pancharatnam-Berry (PB) phase in its left- and right-handed circular polarization components,

9 $$|LP\rangle =\frac{1}{\sqrt{2}}({e}^{-im\theta }|L\rangle +{e}^{im\theta }|R\rangle )$$

where $m=3$ for the three-petal resonator and $m=1$ for the SRR. By spatially varying the orientation of the resonators, continuous but opposite phase variations are expected for the left- and right-handed circular polarization THz field components. Recent experimental work has demonstrated that a linear spatial profile of the PB phase by successively rotating the orientation of SRR^208^ (Fig. 9e) and three-petal^207^ resonators enabled the spatial separation of the left- and right-handed circular polarization components over the entire frequency range of the generated THz radiation. This ultrabroad bandwidth is in marked contrast to the very narrow bandwidth attainable by implementing PB THz metasurfaces to manipulate the post-generated THz radiation^209^. It has been notoriously difficult to directly generate broadband circularly polarized THz radiation. Efforts have been ongoing to develop a THz circular polarizer or linear-to-circular polarization converter, but suffer either from limited bandwidth up to one octave^210,211^ (still much narrower than the bandwidth of typical THz pulses) or being bulky and difficult to deploy^212^. Note that with the fixed phase gradient in the nanoplasmonic metasurfaces, the deflection angle depends on the frequency following the generalized Snell’s law of refraction^213^. This angular dispersion is similar to that in a prism or a grating, so it could be advantageous for certain spectroscopy applications. However, it is undesirable in THz time-domain spectroscopy or applications where broadband excitation with circularly polarized THz radiation is required.

The efficient generation of THz radiation from arrays of nanoplasmonic resonators with appropriate symmetry considerations and spatial phase profiles has opened a new pathway for spatiotemporally shaping the emitted THz radiation^204,207,214^. In order to make this research direction more fruitful, further investigation of the underlying fundamental physics is required. For instance, recent experimental works have revealed that the coupling to the epsilon-near-zero (ENZ) mode in ITO films can have a significant influence on the efficiency of THz generation, and even allow the efficient generation of THz radiation at $y$-polarized excitation in SRRs^208,215^, which was otherwise negligible in the case of no coupling with the ITO ENZ mode^200^. Enhanced THz emission was also observed in dielectric metasurfaces made of GaAs and InAs^216,217^, revealing the significant role of surface and bulk second-order nonlinearities instead of drift currents caused by the surface field or carrier gradient (photo-Dember effect) present in bulk crystals. Thus, we may expect that the THz emission could be greatly enhanced if appropriate materials (ENZ materials, III–V semiconductors and their quantum well structures, graphene, 2D semiconductors, etc.) are integrated to form hybrid nanoplasmonic systems with broken symmetries, further unveiling novel THz generation mechanisms and endowing versatile functionalities for THz applications.

### One-dimensional semiconductor nanowires and carbon nanotubes

**Fig. 10:**
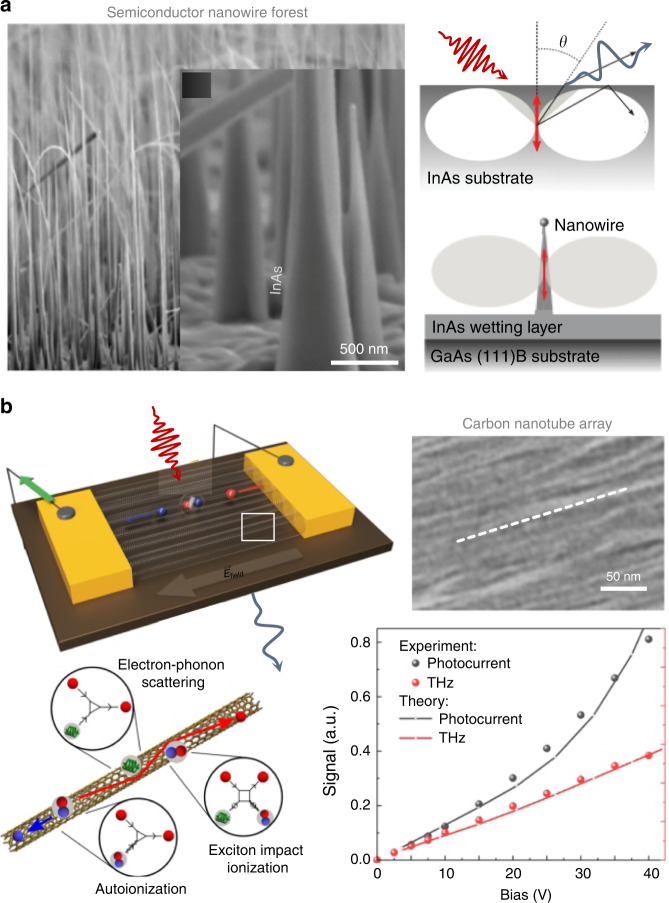
Ultrafast dynamics in 1D materials. **a** Semiconductor (InAs) nanowire forest with enhanced THz outcoupling relative to escape-cone-limited bulk InAs. **b** Exciton dynamics in chirality-enriched semiconducting carbon nanotube array. Superlinear bias dependence for the photocurrent readout indicates the long-timescale exciton multiplication (not present in the ultrafast THz emission signal). Panel (**a**) adapted with permission from ref. ^223^ © 2011 American Physical Society. Panel (**b**) adapted from ref. ^229^ © 2020 American Chemical Society under the terms of CC-BY. Panel (**b**) inset micrograph (nanotube array) adapted with permission from ref. ^230^ © 2016 Nature Publishing Group

While THz applications of 1D systems have already been reviewed extensively^218–220^, we briefly highlight recent insights from THz emission spectroscopy on the ultrafast dynamics in semiconductor nanowires and carbon nanotubes. Greater-than-bulk THz emission efficiencies have been demonstrated in a variety of semiconductor nanowire systems (including Si, GaAs, InAs, and InN), with improved light trapping in dense nanowire forests (Fig. 10a) and resonant leaky modes in highly-ordered arrays increasing the absorption and corresponding THz currents^221^. The photo-Dember effect arising from unequal electron-hole diffusion rates is a common source of charge currents in nanowires^31,222–224^, often in addition to or in competition with junction fields^221,222^, depending on doping type. Impulsive THz plasmonic excitations have also been shown to enhance the THz radiation from Si^225^ and InAs^223^ nanowires. Most significant across all of these applications is the geometry-enhanced THz outcoupling, with a much greater THz escape cone due to the lower-index effective dielectric environment compared with the bulk. Indeed, the escape cone due to total internal reflection at the bulk semiconductor-air interface is particularly limiting, containing only a small fraction of radiated THz power due to the surface-normal dipole orientation^223^. By contrast, an isolated nanowire behaves as an excellent Hertzian dipole^223,225^ (Fig. 10a).

Carbon nanotubes are high aspect ratio cylinders of graphene with typical microscale lengths and nanoscale diameters, which can be single- or multi-walled and exhibit optical and electronic properties (e.g., bandgaps) that depend sensitively on layer number, chiral indices, and tube diameter^226^. Given these properties, arrays often contain mixtures of metallic and semiconducting nanotubes. Individual nanotubes are nominally uniform along their lengths and within uniformly ordered dense arrays, the Schottky barriers formed between the metallic and semiconducting tubes are randomly oriented, canceling out on average. However, THz emission studies have indicated that variation in spatial ordering across different regions of an array can break this symmetry, leading to nonzero drift currents^227^. By contrast, transient photon-drag-induced longitudinal currents were observed via THz emission in a nanotube array preserving top-bottom symmetry^228^. Transverse current contributions were also clearly observed due to inter-nanotube coupling, which could be eliminated by isolating the nanotubes via polymer coatings^228^. Another recent THz emission study of a symmetric, single-walled carbon nanotube photoconductive device (Fig. 10b) revealed more detailed excitonic and free carrier dynamics^229^, enabled by the high-quality, chirality-enriched aligned nanotube array^230^. Sub-picosecond spontaneous exciton dissociation was observed in this study, despite the very large ($\ge$ 100 meV) nanotube exciton binding energies, followed by free-carrier impact generation of additional exciton population (exciton multiplication).

### Two-dimensional materials, heterostructures, and hybrid systems

As a highly interface-sensitive spectroscopy, it is unsurprising that THz emission has been well utilized in 2D material studies^28,231–233^. Since the discovery of graphene^234^ in 2004, a variety of 2D van der Waals materials have emerged as versatile platforms for integrated microelectronic and nanophotonic devices^235–237^. Though most commonly characterized with applied voltage or photoinduced transport methods, THz emission offers a unique ultrafast viewpoint of the hot carrier and other quasiparticle dynamics in these systems for direct characterization of scattering rates, coupling in layered heterostructures, underlying lattice symmetries, and distortions/disorder. Conversely, the 2D material limit may prove useful for the next generation of THz sources, such as photoconductive switches^238–241^, as well as other THz optoelectronics such as detectors and modulators^233^. While many interfacial dynamics have already been discussed above, here we briefly survey some essential materials and heterostructures in the 2D limit, as well as emerging mixed-dimensional systems, which are a promising route toward designer optoelectronic responses and other material properties.

**Fig. 11:**
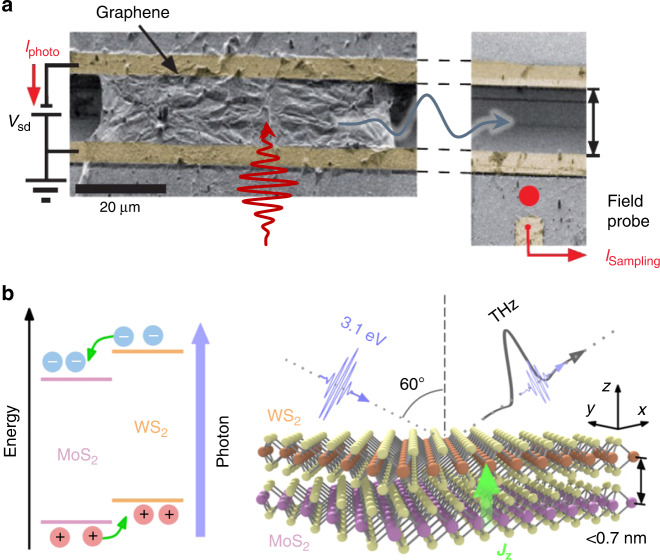
On-chip and free-space THz emission from 2D materials and devices. **a** Suspended graphene on a THz photoconductive strip line. **b** Ultrafast sub-nanometer heterojunction charge flow and THz emission following pulsed excitation, driven by the staggered (type-II) band alignment, with offsets of the same sign between the conduction and valence bands. Panel (**a**) adapted with permission from ref. ^243^ © 2012 the Author(s). Panel (**b**) reprinted with permission from ref. ^258^ © 2019 the Authors

As is customary, we begin by considering graphene, an atomically-thin honeycomb lattice of carbon that exhibits a variety of remarkable electronic and optical properties related, essentially, to the linear energy dispersion within the Dirac cones^242^. Initial studies by Prechtel et al. distinguished THz currents driven by built-in electric fields from much slower photothermoelectric currents at an interface between suspended graphene and a gold strip line^243^ (Fig. 11a), while an even stronger THz photocurrent oscillation upon excitation of the freely-suspended graphene region was attributed to graphene plasmon excitation. Subsequent investigations with off-normal optical illumination of graphene on dielectric substrates^48,244^ and percolated gold films^245^ demonstrated photon-drag-induced currents^246^ and THz radiation. Built-in field and thermoelectric effects were precluded in these contact-free THz emission studies. Recent studies of vertically grown multilayer graphene have further demonstrated an interplay between linear drag currents and helicity-dependent circular photon drag currents, leading to polarization-tunable elliptical THz generation^247^. Competition between these processes was evaluated with respect to the fourth-rank tensors for the D_3*d*_ symmetry of even-layer number Bernal-stacked graphene (AB, ABAB,…) or rhombohedral graphene (ABCABC…) and the D_3*h*_ symmetry of odd-layer-number Bernal graphene. While the photon drag response is present even for nominally centrosymmetric monolayer graphene (${D}_{6h}$) due to directionality induced by the photon momentum, it was shown that the simultaneous out-of-plane ${\mathscr{P}}$-breaking and plasmonic field enhancement of a rough gold film substrate can lead to considerable surface optical rectification^245^.

Recently, a light-induced anomalous Hall current was observed in graphene exposed to circularly polarized light^248^. In this system, the helicity of the optical field breaks ${\mathscr{T}}$ symmetry and opens an intensity-dependent topological Floquet band gap^249^, leading to a Chern insulating state with protected edge transport. A corresponding helicity-dependent THz (few-picosecond) transverse current was generated under a ${\mathscr{P}}$-breaking applied voltage, as read out via a photoconductive switch to demonstrate the light-induced Hall effect. The femtosecond optical excitation allowed for strong driving fields with incident fluences approaching 1 mJ⋅cm^-2^ and tens-of-meV gap openings^248^. This study not only experimentally verified the presence of such light-induced topological states, but also highlighted opportunities to utilize on-chip modalities of THz emission/photocurrent spectroscopy to study quantum and light-induced material properties.

Layered semiconductors such as transition metal dichalcogenides (TMDs), black phosphorus, and some halide perovskites are another, much broader class of 2D materials that have attracted significant attention in the past decade for their layer- or field-dependent optical-range bandgaps, strongly spin-orbit split conduction bands, and corresponding valleytronic applications^236,250,251^. The versatility of integration and engineering of physical properties by stacking different 2D layers^237^ further underpin their usefulness. Prior to the discovery of 2D semiconductors, 2D quantum wells realized in thin ($\sim$10 nm) layers of a low-gap material (e.g., GaAs) sandwiched by higher-gap (e.g., AlAs or Al_x_Ga_1-x_As) were the original designer layered heterostructures, with thickness-, composition-, and layering-dependent confined energy levels, coupling, and optoelectronic properties^14^. Ultrafast single-well electron-hole and exciton polarization dynamics^252^, multi-well resonant tunneling dynamics^253^, and superlattice Bloch oscillations^254,255^ have been explored in these systems via THz emission spectroscopy under different bias voltages. While such systems remain an active area of research^256,257^, atomically-thin semiconductors have become the major frontier in recent years^28,232,236^.

Even so, THz emission studies in the 2D limit of layered semiconductors remain limited^241,258–261^. Competition between in-plane shift current and out-of-plane drift/depletion currents was demonstrated for above-bandgap excitation of few-layer WSe_2_, leading to elliptically-polarized THz radiation that could be tuned by the incident laser polarization and relative sample angle^261^. For above-bandgap excitation of MoSe_2_, by contrast, the suppression of out-of-plane currents in the few-layer limit was demonstrated with a dominant shift current contribution for bilayer samples but a dominant surface depletion drift current in thicker samples^260^. Perhaps the most intriguing THz emission result in the 2D TMD limit thus far has been the observation of sub-nanometer-scale interfacial currents between millimeter-scale MoS_2_ and WS_2_ monolayers grown via chemical vapor deposition^258^. In this study, a type-II band alignment with the valence band maximum in the WS_2_ layer and conduction band minimum in the MoS_2_ layer drove the charge transfer from MoS_2_ to WS_2_ (Fig. 11b), with transfer efficiencies approaching 100%. With standard electro-optic sampling, the large THz signal-to-noise ratio underscores the remarkable sensitivity of THz emission spectroscopy down to angstrom-scale currents.

Finally, opportunities for designer symmetries and dynamics in hybrid systems have been demonstrated in several recent studies. The combination of a 2D material with lateral patterning of nanostructures (especially plasmonic metals) and metasurfaces is relatively straightforward to achieve with existing transfer and lithography techniques, while also providing a high level of versatility in designing local symmetries and light-matter interactions. Such systems have already been extensively explored for optoelectronics, but primarily at the level of enhanced absorption^235^, including purely photonic Purcell enhancements or plasmon “sensitized” charge transfer from the metal. However, recent insights from plasmonic systems^158^, as considered above, suggest much more extensive possibilities for control over momentum and spin degrees of freedom. Valley-polarized charge transfer, for instance, was observed in ${\mathscr{T}}$-broken light-matter interactions mediated by pseudo-chiral gold nanostructures on monolayer MoS_2_, with DC valley Hall currents demonstrated under bias voltages^262^, though details of the suggested spin-selective plasmonic excitation remain to be clarified. Bias-free current generation has been demonstrated in hybrid gold-graphene systems with ${\mathscr{P}}$-broken nanostructures of different designer in-plane spatial symmetries^263,264^. These effectively DC responses were generated by a well-known photothermoelectric effect at the gold-graphene interfaces^265,266^, with net directionality due to the oriented plasmonic hot spots. While nascent, these studies demonstrate opportunities for control over many charge carrier degrees of freedom and their THz dynamics, tailored by materials selection and their intrinsic symmetries and physical properties, as well as patterned nanoscale structural symmetries.

## Summary and outlook

A wide variety of materials and mechanisms have been investigated using THz emission spectroscopy, in many instances revealing dynamics and corresponding locally broken symmetries that would be difficult or impossible to observe with other techniques. As a starting point, the mere presence of THz emission reveals a broken discrete symmetry (${\mathscr{P}}$, ${\mathscr{T}}$, or ${\mathscr{P}}{\mathscr{T}}$). The sensitivity of the THz amplitude, phase, polarization, Poynting vector, and frequency spectrum to the corresponding parameters of the input optical pulse may then be utilized to map out intrinsic point group structure, extrinsic geometrical structuring, and hybrid junctions of material systems down to the nanoscale. This clarifies the various vanishing and nonvanishing elements of the nonlinear constitutive tensors connecting induced photoresponses with incident optical fields. Beyond these underlying symmetry properties, the detailed dynamics of various quasiparticle (e.g., hot carrier, Cooper pair, magnon) degrees of freedom and coupling thereof can be reconstructed in the time and frequency domains.

While THz emission spectroscopy has proven useful for exploring the complex behaviors of bulk quantum materials and interfaces, it remains poised to enable the further discovery of novel physics and phases in many emerging materials, including nanoscale systems. Indeed, opportunities to exploit extrinsic nanoscale structuring for probing hidden properties or inducing new behaviors in these materials remain much less explored. The diverse selection of THz dynamics that have been observed so far in relatively simple nanostructured metals and semiconductors suggests that an even broader variety of novel behaviors are likely to emerge in low-dimensional (including artificially structured) strongly correlated, magnetic, and topological material systems. Toward these directions, new capabilities for tip-based THz emission mapping will provide higher spatial resolution, into the deeply sub-diffraction nanoscale regime^192–194^, while new detector technologies will provide greater sensitivity and bandwidth for higher temporal resolution, into the tens-of-femtosecond range^18^. Furthermore, THz emission spectroscopy remains underutilized for observing external photocurrent dynamics in perturbative multiphoton and strong-field tunneling emission processes. In nanostructured systems, such photoemission and corresponding THz emission is often dominated by few-nanometer hot spot regions. Thus, nanostructured and particularly hybrid plasmonic systems serve as another promising route for probing nanoscale physics and ultrafast light-matter interactions via THz emission, complementing tip-based methods. Finally, the on-chip modality of THz emission spectroscopy with THz waveguides and photoconductive switches represents another nascent capability for studying sub-diffraction nano–microscale THz emission in 2D^243,248^, 1D^31^, and nanostructured^188^ material systems. Recent progress in this area suggests many fruitful opportunities on the near horizon for investigating 2D heterostructures, Moiré superlattices, mixed-dimensional systems, and hybrid nanostructured materials with the unique perspective on symmetries and dynamics offered by THz emission spectroscopy.
